# BCTI: a Bayesian network-based method for revealing critical transitions in complex biological systems

**DOI:** 10.7717/peerj.20860

**Published:** 2026-02-13

**Authors:** Yuyan Tong, Renhao Hong, Na Yang, Pei Chen, Hao Peng, Hui Tang, Rui Liu

**Affiliations:** 1 School of Mathematics, South China University of Technology, Guangzhou, Guangdong, China; 2 School of Mathematics, Foshan University, Foshan, China

**Keywords:** Dynamic network biomarker (DNB), Critical transition, Disease progression, Gene regulatory network (GRN), Bayesian network structure learning

## Abstract

**Background:**

The identification of critical states during disease progression is essential yet challenging for preventing disease deterioration and developing precision therapies. Traditional methods often rely on the dynamic feature of coordinated molecular variation to provide early-warning signals of impending critical transitions. However, these methods typically overlook the causal relationships among variables, potentially limiting their interpretability in uncovering underlying molecular regulatory mechanisms.

**Methods:**

With the rapid advancement of sequencing technologies and the surge in high-throughput data, we propose Bayesian Critical Transitions Inference (BCTI), inspired by the time-varying nature of gene regulatory networks. BCTI integrates mutual information and structural equation models to qualitatively capture dynamic changes in network topology and quantitatively evaluate system states through a network scoring mechanism, thereby enabling the efficient and robust dual detection of early-warning signals associated with critical transitions in disease progression.

**Results:**

The proposed BCTI was validated by a series of applications on simulated and real datasets of complex biological systems. BCTI achieved superior or comparable accuracy to benchmark methods in inferring gene regulatory networks (GRNs) and detecting critical states. All the results demonstrate the high effectiveness of the proposed method in analyzing time-course/stage-course high-dimensional expression data, providing new insights into precision medicine for clinical applications and the underlying regulatory mechanisms of biological systems.

**Conclusions:**

The proposed method enables effective detection of critical transitions and reveals dynamic regulatory mechanisms in complex biological systems, demonstrating strong potential for applications in systems biology, precision medicine, and the exploration of key molecular regulation driving disease progression and development.

## Introduction

**Figure 1:**
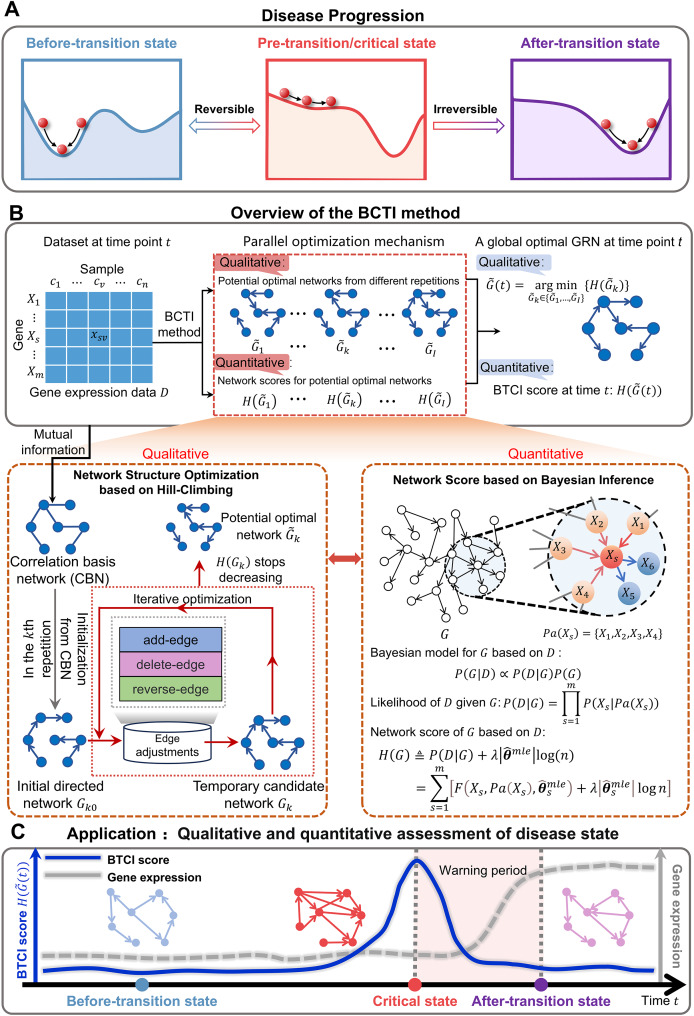
Schematic illustration for uncovering a pre-transition state using BCTI. (A) Disease evolution can be broken down into three distinct states: a stable before-transition state, a pre-transition/critical state, and another stable after-transition state. The pre-transition state is distinguished by diminished resilience and heightened susceptibility and is often reversible back to the before-transition state through suitable intervention. (B) Given gene expression data with genes and samples, BCTI employs a parallel optimization mechanism to qualitatively and quantitatively output the global optimal network at the current time. The bottom-left panel qualitatively optimizes the network structure based on hill-climbing, while the bottom-right panel quantitatively defines network scores based on Bayesian inference to guide the network optimization. (C) The significant change in BCTI scores indicates a critical state of a complex disease, where the BCTI score at the time point is the score corresponding to the current global optimal GRN.

The progression of complex diseases, such as chronic inflammation and various cancers, can be regarded as a nonlinear dynamic process, which is generally divided into three states: before-transition state, pre-transition/critical state and after-transition state (Chen et al., 2012) (Fig. 1A and Figs. S1–S3). The before-transition state is a relatively healthy state that is stable with strong resilience, whereas the after-transition state is another stable state. The critical state represents a tipping point before disease deterioration, where timely intervention may reverse the system to the before-transition state (Stead et al., 2010). However, identifying the critical state remains challenging due to the subtle molecular and phenotypic differences between it and the before-transition state (Liu et al., 2014). Recently, the dynamic network biomarker (DNB) (Chen et al., 2012; van de Leemput et al., 2014; Aihara et al., 2022; Chen et al., 2019) has been proposed to quantitatively signal the imminent critical transitions in biological systems, by leveraging a dominant group of biomolecules that exhibit significant fluctuations and strong collective behaviors (Note S1). DNB and its extensions have made significant contributions to the identification of tipping points from a network perspective (Xie, Peng & Li, 2024; Sun et al., 2020). To further enhance the interpretability and mechanistic understanding of critical transitions, there is an urgent need to incorporate causal modeling, enabling deeper insights into the underlying molecular regulatory mechanisms.

Against this background, gene regulatory networks (GRNs), as graph-based models that capture regulatory relationships between genes within cells, offer a new perspective for uncovering causal mechanisms in disease systems (Liu et al., 2022). GRNs represent genes as nodes and regulatory interactions as edges, enabling systematic modeling of gene–gene relationships that drive cellular responses (Mercatelli et al., 2020; Harwood & Moszer, 2002). Existing studies have shown that GRNs are not static, their network topology and composition may change dynamically over time (Meng et al., 2010; Pan & Zhang, 2024). Capturing such temporal changes in regulatory mechanisms is essential for signaling and understanding critical transitions in biological system states. For instance, wild-type *TP53* activates *BAX* to induce apoptosis early in tumor development, but this regulation is lost in later stages due to *TP53* mutation, allowing tumor cells to evade apoptosis (Schuyer et al., 2001). Notably, tracking these regulatory changes provides insights into disease-driving mechanisms and supports the discovery of potential therapeutic targets.

With the rapid advancement of high-throughput sequencing technologies, vast amounts of biomolecular expression data have been generated, in turn driving the development of a variety of GRN inference methods. These methods primarily include correlation and information-theoretic approaches, Boolean network approaches, regression models, and differential equation-based models (Mercatelli et al., 2020). Among them, correlation and information-theoretic methods identify statistical dependencies between gene expression profiles (Soranzo, Bianconi & Altafini, 2007); Boolean network approaches simplify gene states into binary variables and model regulatory interactions using logical rules (Lim et al., 2016); regression models treat GRN inference as a feature selection problem to identify potential key regulators (Omranian et al., 2016); and differential equation-based approaches simulate dynamics of gene expression, though they often rely on steady-state assumptions to produce reliable results (Äijö & Lähdesmäki, 2009). Although the aforementioned methods are relatively mature in terms of network construction, they primarily focus on static network inference, making it difficult to effectively identify critical states during disease progression or to reveal the underlying mechanisms of functional disruption in complex diseases from the perspective of dynamic network reconstruction.

To address the above challenges, we propose Bayesian Critical Transitions Inference (BCTI), a unified framework that models both the causal structure and temporal dynamics of gene regulatory networks, enabling simultaneous qualitative and quantitative detection of critical transitions of systems (Figs. 1B,1C). Specifically, BCTI first constructs a correlation basis network (CBN) using mutual information and then infers causal relationships between variables *via* a Bayesian network search guided by the minimum description length (MDL) principle. By tracking the temporal evolution of both network topology and corresponding model scores, BCTI captures critical transitions with structural precision and quantitative clarity. To evaluate the effectiveness and robustness of BCTI, we applied it to several benchmark datasets, including the Dialogue on Reverse Engineering Assessment and Methods (DREAM) challenge (Marbach et al., 2010), the *Saccharomyces cerevisiae* synthetic *in vivo* assessment of reverse-engineering and modeling assessment (IRMA) network (Cantone et al., 2009), the *E. coli* SOS repair network dataset (Ronen et al., 2002) and a sixteen-node regulatory network (Liu, Chen & Chen, 2020) datasets. In addition, BCTI was applied to real-world datasets, including bulk RNA-seq data from colon adenocarcinoma (COAD), lung adenocarcinoma (LUAD), and thyroid adenocarcinoma (THCA) in The Cancer Genome Atlas (TCGA), as well as single-cell transcriptomic data from human embryonic lung development (Sountoulidis et al., 2023). By combining qualitative analysis of regulatory network topology with quantitative assessment of system dynamics, BCTI successfully identified critical states in diverse datasets, demonstrating broad applicability to a wide range of biological systems.

## Materials and Methods

To investigate the underlying causal mechanisms of critical state transitions in complex biological systems, we employ Bayesian networks as a foundational modeling framework. Bayesian networks provide a principled probabilistic approach for inferring directed regulatory relationships among genes, going beyond simple statistical associations. Moreover, they enable score-based structure learning, which systematically balances model fit and complexity. These features make Bayesian networks particularly suitable for capturing dynamic changes in gene regulatory architectures associated with critical transitions.

### Bayesian network fundamentals

A Bayesian network is a probabilistic graphical model that represents conditional dependencies among variables (*e.g*., gene expression levels) using a directed acyclic graph (DAG). In this framework, nodes correspond to genes, and directed edges represent potential regulatory relationships. The goal of Bayesian network structure learning is to identify the optimal network $\tilde G$ that best explains the observed data $D$, *i.e*., the network that maximizes the posterior probability $P\left( {G|D} \right)$ (Fig. 1B). According to Bayes’ theorem, the posterior probability of a network $G$ given data $D$ is:

(1) $$\matrix{ {P\left( {G|D} \right) \propto P\left( {D|G} \right)P\left( G \right)}}\!.$$

Notably, maximizing the posterior probability is equivalent to maximizing the product of the likelihood $P\left( {D|G} \right)$ and the prior $P\left( G \right)$:

(2) $$\matrix{ {\tilde G = \mathop {{\rm arg\; max}}\limits_G P\left( {G|D} \right) = \mathop {{\rm arg\; max}}\limits_G P\left( {D|G} \right)P\left( G \right)}. \cr }$$

For a given Bayesian network structure $G$, the likelihood can be factorized as:

(3) $$\matrix{ {P\left( {D{\rm |}G} \right) = \mathop \prod \limits_{s = 1}^m P\left( {{X_s}|Pa\left( {{X_s}} \right)} \right) = \mathop \prod \limits_{s = 1}^m \mathop \prod \limits_{j = 1}^n P\left( {{x_{sj}}|Pa\left( {{X_s}} \right)} \right)}\cr }$$

where ${X_s}$ represents the random variable corresponding to the expression level of gene ${g_s}$, and $Pa\left( {{X_s}} \right)$ represents the parent nodes (regulators) of ${X_s}$ in $G$. The term ${x_{sj}}\;$ refers to the observed expression level of gene ${g_s}$ in sample $j$. This factorization leverages the conditional independence properties encoded in the DAG, allowing efficient modeling of high-dimensional data.

Bayesian networks provide a powerful framework for modeling the causal relationships among genes. However, traditional Bayesian network models are primarily designed for discrete variables, whereas gene expression data are inherently continuous. To address this mismatch, we adopt structural equation models (SEMs) as continuous-variable counterparts within the Bayesian framework.

SEMs model each gene as a linear combination of its parent genes and a random noise term. This formulation allows us to leverage the causal interpretability of Bayesian networks while accommodating the continuous nature of high-throughput transcriptomic data. Moreover, SEMs allow for the estimation of model fit *via* residuals, which directly feed into our network scoring criterion.

For continuous gene expression data, the conditional probabilities $P\left( {{X_s}|Pa\left( {{X_s}} \right)} \right)$ can be modeled using SEMs, which represent each variable ${X_s}$ as a linear combination of its parent nodes $Pa\left( {{X_s}} \right)$ with an added noise term:

(4) $$\matrix{ {{{x}_s} = {{\boldsymbol{\theta }}_s}{{{x}}_{Pa\left( {{X_s}} \right)}} + {{\epsilon }_s}}, \cr }$$

where ${{x}_s} \in {{{\mathbb R}}^{1 \times n}}$ is the expression vector of ${X_s}$, ${{x}_{Pa\left( {{X_s}} \right)}} \in {{{\mathbb R}}^{p \times n}}$ represents the expression matrix of $Pa\left( {{X_s}} \right)$, where $p$ is the number of the parents, ${{\boldsymbol{\theta }}_s} \in {{{\mathbb R}}^{1 \times p}}$ is the regression coefficient vector to be estimated, and ${{ \epsilon }_s} \in {{{\mathbb R}}^{1 \times n}}$ is a Gaussian noise vector.

Furthermore, we develop a network score, *i.e*., $H$ score, based on the minimum description length (MDL) principle in information theory to guide the Bayesian network structure learning, which considers both the goodness-of-fit to the data and the network’s complexity:

(5) $$\matrix{ {H\left( G \right) = \mathop \sum \limits_{s = 1}^m \left[ {F\left( {{X_s},Pa\left( {{X_s}} \right),{\widehat{\boldsymbol{\theta}} }_s^{mle}} \right) + \lambda \left| {\widehat{\boldsymbol{\theta}}_s^{mle}} \right|\log n} \right]}, \cr }$$

where:

(6) $$\matrix{ {F\left( {{X_s},Pa\left( {{X_s}} \right),{\widehat{\boldsymbol{\theta}}}_s^{mle}} \right) = \mathop \sum \limits_{v = 1}^n {{\left[ {{x_{sv}} - {\widehat{\boldsymbol{\theta}}}_s^{mle}{{{x}}_{Pa\left( {{X_s}} \right)v}}} \right]}^2}}, \cr }$$

${{{x}}_{Pa\left( {{X_s}} \right)v}} \in {{{\mathbb R}}^{p \times 1}}$ represents the expression vector of the parents $Pa\left( {{X_s}} \right)$ in the $v$th sample, ${\widehat{\boldsymbol{\theta}}}_s^{mle} = \mathop {\arg \min }\limits_{{{\theta }}_{s}} F\left( {{X_s},Pa\left( {{X_s}} \right),{{\boldsymbol{\theta }}_s}} \right) \in {{{\mathbb R}}^{1 \times p}}$ is the maximum likelihood estimate of the parameter ${{\theta }_s}$ using least squares when $F\left( {{X_s},Pa\left( {{X_s}} \right),{\boldsymbol{\theta }}_{s}} \right)$ is the minimum, and $\left| {{\widehat{{\boldsymbol \theta}}}_s^{mle}} \right|$ is the number of to-be-estimated parameters in ${\hat {\boldsymbol \theta} }_s^{mle}$ (in this study, it is the number of parents, *i.e*., $\left| {{\widehat{\boldsymbol{\theta}}}_s^{mle}} \right| = \left| {{Pa}\left( {{X_s}} \right)} \right|$). Noteworthily, $\left[ {F\left( {{X_s},Pa\left( {{X_s}} \right),{\hat \theta }_s^{mle}} \right) + \lambda \left| {{\hat \theta }_s^{mle}} \right|\log n} \right]$ represents the evaluation score for the local subnetwork consisting of ${X_s}$ and its parents, called a local subnetwork score, while $H\left( G \right)$ represents the sum of the local subnetwork scores, with each subnetwork corresponding to a specific gene in $G$. Here, $F\left( {{X_s},{Pa}\left( {{X_s}} \right),{\widehat{{\boldsymbol \theta}}}_s^{mle}} \right)$ measures the goodness of fit to the data, and $\lambda \left| {{\widehat{\boldsymbol{\theta}}}_s^{mle}} \right|\log n$ quantifies the network complexity. The regularization parameter $\lambda$ controls the trade-off between the goodness of fit and the network complexity.

### Workflow of BCTI

Given gene expression data with $m$ genes from different time points, to investigate the dynamic changes in gene regulatory networks and uncover critical states in complex biological systems, the algorithm of BCTI is proposed to simultaneously perform quantitative and qualitative analyses of system states from a network-level perspective:

*Step 1. Construct the undirected correlation basis network (CBN) at each time point*.

In this study, mutual information is a measure used to quantify the correlation between two random variables, capturing both linear and nonlinear dependencies (Kraskov, Stögbauer & Grassberger, 2004). For random variables ${X_i}$ and ${X_j}$, $i,j \in \left\{ {1,2, \ldots ,m} \right\}$, the mutual information between ${X_i}$ and ${X_j}$ is defined as the following form:

(7) $$\matrix{ {I\left( {{X_i},{X_j}} \right) = \mathop \sum \limits_{x \in {X_i},y \in {X_j}} p\left( {x,y} \right){\rm log}\displaystyle{{p\left( {x,y} \right)} \over {p\left( x \right)p\left( y \right)}}},\cr }$$

where $p\left( x \right)$ (or $p\left( y \right)$) represents the probability that the value of the variable ${X_i}$ (or ${X_j}$) is equal to $x$ (or $y$), and $p\left( {x,y} \right)$ represents the joint probability of ${X_i}$ and ${X_j}$. To improve computational stability and efficiency, we followed a previous study (Zhao et al., 2016) and simplified Eq. (7) under the assumption that the variables follow Gaussian distributions:

(8) $$\matrix{ {I\left( {{X_i},{X_j}} \right) = - \displaystyle{1 \over 2}\log \left( {1 - \rho _{ij}^2} \right)}, \cr }$$

where ${\rho _{ij}}$ is the Pearson correlation coefficient between ${X_i}$ and ${X_j}$. To prevent numerical instability when ${\rho _{ij}}$ approaches 1, a small constant ( $\varepsilon = {10^{ - 5}}$) was added inside the logarithmic term during the computation:

(9) $$\matrix{ {I\left( {{X_i},{X_j}} \right) = - \displaystyle{1 \over 2}\log \left( {1 - \rho _{ij}^{2} + \varepsilon } \right).} \cr }$$

More computational details are provided in Note S2. To construct a CBN that captures adjacency relationships among genes, we set a default threshold $T = 0.1$ to evaluate the relevance between genes, following previous network-based studies (Chaitankar et al., 2009, 2010; Yang et al., 2016; Zhang et al., 2012), where a similar cutoff was used to retain potentially relevant associations while excluding unlikely connections. Specifically, if $I\left( {{X_i},{X_j}} \right) \ge T$, ${X_i}$ is deemed related to ${X_j}$; otherwise, if $I\left( {{X_i},{X_j}} \right) < T$, the mutual information is insufficient to establish relevance, thus ${X_i}$ and ${X_j}$ are treated as independent (Liu et al., 2016), $i,j \in \left\{ {1,2, \ldots ,m} \right\}$. Essentially, the CBN illustrates the potential regulatory relationships between genes but does not indicate the direction of regulation.

*Step 2. Randomly generate an initial DAG*.

To enhance the likelihood of identifying the global optimal GRN, we repeatedly generate different initial DAGs from the undirected CBN structure. Taking the $k$th repetition as an example ( $k \in \left\{ {1,2, \ldots ,I} \right\}$), an initial DAG, conforming to the CBN structure, is randomly generated and denoted as ${G_{k0}}$.

*Step 3. Determine a potential optimal network (* ${\tilde G_k}$*) using a hill-climbing search*.

Letting ${\tilde G_k} = {G_{k0}}$, we first apply the add-edge, delete-edge, and reverse-edge operations to ${G_{k0}}$, generating three sets of adjusted networks, denoted as ${C_1}$, ${C_2}$, and ${C_3}$, respectively (Note: these operations only target the edges identified in the CBN). Next, the network with the lowest $H$ score in each of the sets, called an alternative network, is identified for subsequent analysis and is denoted as follows:

(10) $$\matrix{ {{G_{k1}} = \mathop {{\rm arg\; min}}\limits_{G_{k1}^i \in {C_1}} \left\{ {H\left( {G_{k1}^i} \right)} \right\}}, \cr }$$

(11) $$\matrix{ {{G_{k2}} = \mathop {{\rm arg\; min}}\limits_{G_{k2}^j \in {C_2}} \left\{ {H\left( {G_{k2}^j} \right)} \right\}}, \cr }$$

(12) $$\matrix{ {{G_{k3}} = \mathop {{\rm arg\; min}}\limits_{G_{k3}^q \in {C_3}} \left\{ {H\left( {G_{k3}^q} \right)} \right\}.} \cr }$$

Subsequently, the $H$ scores of these alternative networks are compared to obtain a temporary candidate network, denoted as ${G_k}$:

(13) $$\matrix{ {{G_k} = \mathop {{\rm arg\; min}}\limits_{{G_{kt}} \in \left\{ {{G_{k1}},{G_{k2}},{G_{k3}}} \right\}} \{ H({G_{kt}})\}}. \cr }$$

If the $H$ score of ${G_k}$ is lower than that of ${\tilde G_k}$, then ${\tilde G_k} = {G_k}$. Next, we set ${\tilde G_k}$ as the new initial directed network and continue performing edge adjustments, applying the adjustment that yields the minimum $H$ score to refine ${\tilde G_k}$ and produce a new ${G_k}$. This process is repeated iteratively until the $H$ score of the temporary candidate network no longer decreases, indicating convergence. The ${\tilde G_k}$ after the above procedure is regarded as the final potential optimal network in this repetition.

*Step 4. Determine a global optimal network*.

After obtaining potential optimal networks from different repetitions $\left\{ {{{\tilde G}_1}, \ldots ,{{\tilde G}_I}} \right\}$, we take the network with the minimum $H$ score among them as the global optimal network $\tilde G$, that is:

(14) $$\matrix{ {\tilde G = \mathop {{\rm arg\; min}}\limits_{{{\tilde G}_k} \in \left\{ {{{\tilde G}_1}, \ldots ,{{\tilde G}_I}} \right\}} \{ H({{\tilde G}_k})\}}, \cr }$$

thereby qualitatively and quantitatively describing the system’s network state at the current time point.

*Step 5. Identify critical states based on a dual evaluation mechanism of network scoring and structural stability*.

At each time point $t$, we calculate the $H$ score of the global optimal network $\tilde G\left( t \right)$ (*i.e*., $H\left( {\tilde G\left( t \right)} \right)$) following the above procedures. This score, named BCTI score and denoted by $S\left( t \right)$, provides a quantitative measure of the network’s temporal dynamics and serves as a key indicator for identifying potential critical transitions. In terms of the DNB theory, the system approaching a critical point typically exhibits distinct dynamical changes within a core subnetwork: (i) sharply increased fluctuations (such as standard deviation) in the expression of key genes; (ii) markedly enhanced coordinated variation (such as Pearson correlation coefficient) among key genes. In our method, these two features are reflected in the dimensions of network scoring and structural stability, respectively, and jointly drive the identification of critical states.

Specifically, in the dimension of network scoring, it is demonstrated that the defined scoring function $H\left( \cdot \right)$ is theoretically derived to be positively correlated with the variance of gene expression (Note S3). Due to the markedly increased gene expression fluctuations near the critical state, the BCTI score rises correspondingly and can therefore serve as a quantitative indicator of declining system stability. To statistically identify the effective signal, we apply a one-sample *t*-test to the BCTI scores. The time point $T = t$ is defined as a critical point if there is a significant difference between the current BCTI score $S\left( t \right)$ and the mean value of a vector $\left( {S\left( 1 \right),S\left( 2 \right), \ldots ,S\left( {t - 1} \right)} \right)$ ( $P < 0.05$). More details are provided in Note S4. In the dimension of structural stability, BCTI employs structural equation models to reconstruct gene regulatory networks. When the system is at the critical point, markedly enhanced coordinated variation among key genes leads to severe multicollinearity, which substantially increases the variance of regression coefficient estimates during structural equation model fitting (Grewal, Cote & Baumgartner, 2004). It disrupts the stable inference of causal relationships and leads to instability in network reconstruction, thereby reducing the accuracy of identifying regulatory relationships. Therefore, the combination of elevated BCTI scores and decreased network stability provides a dual criterion for identifying an impending critical transition and helps distinguish true system transitions from isolated anomalies.

## Results

The BCTI algorithm and related definitions are presented in the Materials and Methods section. To demonstrate the effectiveness of BCTI, it was tested on several simulated and real datasets. On one hand, to benchmark the performance of BCTI on the GRN inference, we compared it with several well-known methods on the aforementioned datasets, including GENIE3 (Huynh-Thu et al., 2010), GENIMS (Wu et al., 2016), GNIPLR (Zhang, Chang & Liu, 2021), KBoost (Iglesias-Martinez, De Kegel & Kolch, 2021), NARROMI (Zhang et al., 2013), NIMEFI (Ruyssinck et al., 2014) and PLSET (Guo et al., 2016). Detailed information about the benchmark methods and the metrics used to evaluate GRN inference performance is provided in Note S5. Additional details on the datasets can be found in Tables S1 and S2. On the other hand, to validate the critical states identified in the TCGA datasets, we conducted Kaplan–Meier (log-rank) survival analysis to compare the prognostic outcomes of before-transition and after-transition samples. Additionally, we performed biological functional analysis to validate the critical state identified in the lung development dataset.

### Efficiency of BTCI on inferring causal relationships in GRNs

To evaluate the performance of BCTI in inferring causal relationships within GRNs, we compared it against several methods, including GENIE3-RF-sqrt, GENIE3-ET-sqrt, GENIMS, GNIPLR, NARROMI, NIMEFI, and PLSET, using benchmark datasets such as the DREAM network, the IRMA network, and the SOS DNA repair experimental dataset. For the DREAM and IRMA networks, BCTI successfully inferred most of the edges in the gold standard networks, achieving 85.56% and 85.00% accuracy, respectively, outperforming other methods (Tables S3 and S4). On the SOS DNA repair dataset, BCTI correctly inferred 14 out of 24 regulatory edges and achieved the highest true positive (TP) count, indicating strong performance in recovering the underlying regulatory structure (Table S5). These results demonstrate the effectiveness and robustness of BCTI in inferring gene regulatory networks across both simulated and experimental datasets, highlighting its ability to achieve accurate and reliable causal relationship inference under diverse data conditions. Additional computational details are provided in Tables S3–S5.

### Accurate and robust critical-state detection performance of BCTI compared with existing methods

To examine the ability of BCTI to identify critical transitions, we employed a simulation framework based on a previously published gene regulatory network model that has been widely used to study bifurcation-driven dynamics in biological systems (Foo, Kim & Bates, 2018; Chen et al., 2015). In this framework, regulatory interactions are described using Michaelis–Menten–type kinetics and the system evolution is governed by stochastic differential equations. Following this established simulation setting, system dynamics were generated by gradually varying a control parameter ( $q$) within the range of −0.3 to 0.3, which drives the system toward a bifurcation point. The resulting simulated time-series data were then used to evaluate the effectiveness of BCTI in detecting the onset of critical states. Detailed model formulations and parameter settings are provided in Note S6.

**Figure 2:**
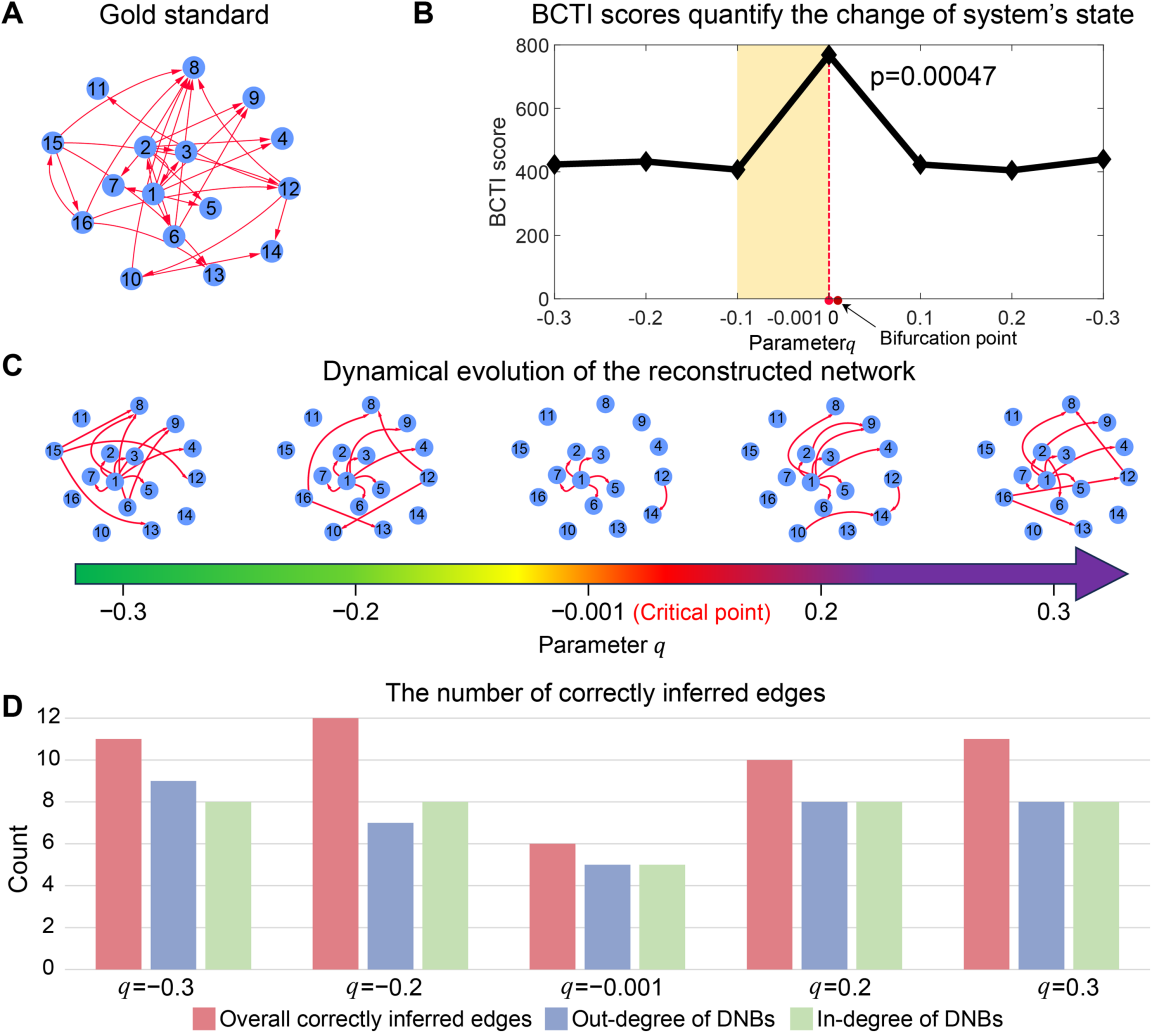
Performance of BCTI illustrated by a 16-node simulated network model. (A) The gold standard simulated network. (B) The BCTI curve quantifies the change in the system’s state. It is evident that the BCTI score suddenly increases near the critical point. (C) The dynamic evolution of the reconstructed network reveals a notable transition in structure and alignment with the gold standard network near the critical point. (D) The consistency between the overall network and the gold standard, as well as the alignment of in-degrees and out-degrees of key nodes, both reach a minimum at the critical state, signaling the system’s approach to a critical transition.

As depicted in Fig. 2B, the BCTI score exhibited an abrupt increase as the system approached to the bifurcation point ( $q = 0$), indicating an impending critical point. To visually emphasize the dynamic differences between the before-transition and critical states, we presented the evolution of the network reconstructed by BCTI (Fig. 2C). Notably, when the system was far from the critical point (such as $q = - 0.2$), the reconstructed network exhibited notable overlap ( ${\rm TP} = 12$) with the gold standard network, highlighting the system’s stable state and demonstrating BCTI’s efficacy at such a stage (Fig. 2D). However, as the system was near the critical point ( $q = - 0.001$), there was a notable decrease in the overlap ( ${\rm TP} = 6$) between the reconstructed network and the gold standard network, indicating an impending transition of the system’s state (Fig. 2D). To further underscore the robustness of BCTI, we conducted comparative experiments under varying noise levels against several benchmarking methods (Chen et al., 2012; Peng et al., 2022), including the node-based, direct interaction network-based divergence (DIND), and DNB methods (Fig. S5). Under noise-free conditions (noise strength $\sigma = 0$), all methods were able to indicate the tipping point. However, as the noise strength increased (*e.g*., $\sigma = 0.5$ or $\sigma = 1.5$), the node-based, DIND, and DNB methods exhibited substantial instability or even failed to identify the critical transition effectively. In contrast, BCTI consistently exhibited its effectiveness and robustness in identifying tipping points, maintaining high sensitivity and producing clear early-warning signals across all tested noise levels. More details for the theoretical background of BCTI and computational results are provided in Note S3 and Table S6. The above numerical experiment validates the reliability and accuracy of BCTI in identifying critical states at the network level.

### Efficiency of BCTI on uncovering underlying gene regulatory mechanisms of cancers

**Figure 3:**
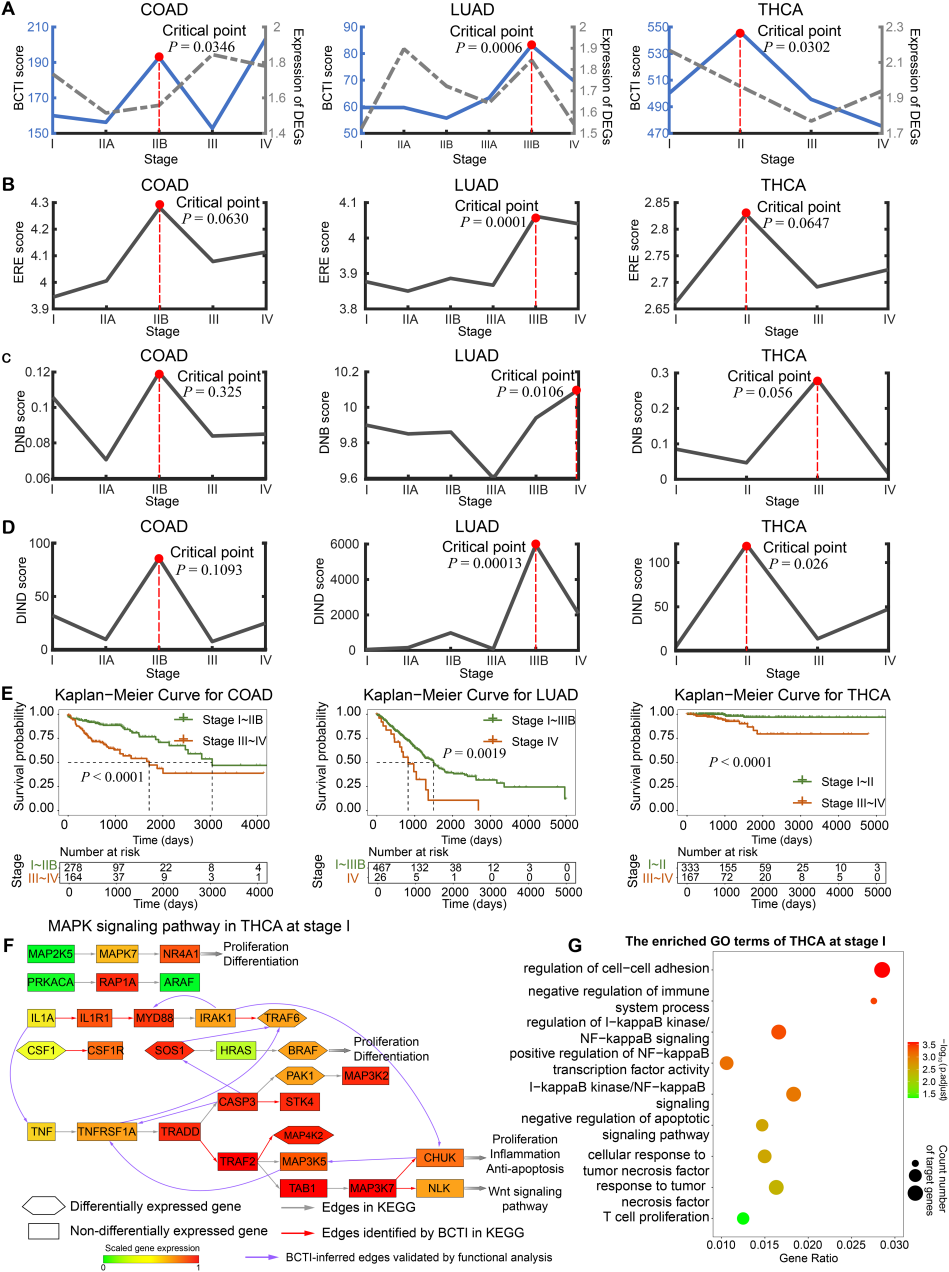
Identifying critical states for tumor deterioration. (A) The performance comparison of BCTI and DEGs in identifying the critical states for different tumor datasets: COAD, LUAD, and THCA. (B and C) The critical-state detection performance of existing methods in different tumor datasets. (E) Survival curves for patients before and after the BCTI-inferred critical stage: COAD, LUAD, and THCA. (F and G) For the BCTI-inferred regulatory relationships, GO functional enrichment results biologically validated their significant association with tumor-related biological processes.

Furthermore, BCTI was applied to three TCGA datasets: COAD, LUAD, and THCA. For each cancer type, we selected biologically relevant pathogenic pathways known to be strongly associated with the disease and extracted key genes from these pathways to reconstruct the gene regulatory networks. In other words, these genes were predefined prior to network inference based on curated biological knowledge. The same set of genes was used across all tumor stages within each cancer type to ensure the comparability of the reconstructed networks. For COAD, the verification network was derived from the pathway hsa04151 (PI3K-Akt signaling pathway) in the Kyoto Encyclopedia of Genes and Genomes (KEGG) database (https://www.genome.jp/kegg/pathway.html). For LUAD, we took the pathway hsa04064 (NF-kappa B signaling pathway) as the verification network due to its tumor promoting role in lung carcinogenesis (Chen et al., 2011). For THCA, the pathway hsa04010 (MAPK signaling pathway) was selected as the verification network, as it has been widely studied and plays a crucial role in thyroid carcinogenesis (Hu et al., 2021). Implementing the procedure outlined in the Methods, we rewired the genetic network within the functional pathways at each tumor stage and calculated BCTI scores to identify the critical states during cancer progression (Fig. 3). On one hand, as shown in Figs. S6–S8, BCTI characterized the dynamic evolution of the rewired regulatory subnetworks in the PI3K-Akt signaling pathway across all five stages, the NF-kappa B signaling pathway across six stages, and the MAPK signaling pathway across four stages, respectively. Overall, BCTI demonstrates strong network reconstruction capabilities by accurately identifying true regulatory edges within pathways during early stages of disease progression. However, its performance declines markedly at certain critical stages. For instance, only two true regulatory edges were detected in stage IIB of COAD ( $TPR = 0.1333$), none were identified in stage IIIB of LUAD ( $TPR = 0$), and only three in stage II of THCA ( $TPR = 0.1250$). Compared to other stages, the notable drop in reconstruction accuracy in these stages suggests the system approaches a critical state. According to the network stability criterion proposed in the Methods section, this phenomenon may arise from enhanced coordinated variation among key genes near the critical state, which increases multicollinearity among variables and consequently disrupts the stable estimation of regulatory relationships in the structural equation models. More computational details are provided in Table 1.

**Table 1:** Network inference performance of BCTI in different cancers. The performance of BCTI in reconstructing gene regulatory networks across different cancer stages in COAD, LUAD, and THCA. Each stage is evaluated using four classification metrics: true positives (TP), false positives (FP), false negatives (FN), and true negatives (TN), with the true positive rate (TPR) calculated accordingly. The identified critical stage for each cancer type is highlighted in bold. Lower TPR values at these critical stages indicate heightened system instability and reduced predictability, serving as early-warning signals.

Datasets	Indices\ Stages	TP	FP	FN	TN	TPR
COAD	I	6	14	9	181	0.4000
	IIA	4	23	11	172	0.2667
	IIB (Critical stage)	**2** ^a^	**19** ^a^	**13** ^a^	**176** ^a^	**0.1333** ^a^
	III	7	19	8	176	0.4667
	IV	2	23	13	172	0.1333
LUAD	I	5	23	7	175	0.4167
	IIA	2	14	10	184	0.1667
	IIB	3	16	9	182	0.2500
	IIIA	2	13	10	185	0.1667
	IIIB (Critical stage)	**0** ^a^	**11** ^a^	**12** ^a^	**187** ^a^	**0** ^a^
	IV	1	15	11	183	0.0833
THCA	I	9	59	15	787	0.3750
	II (Critical stage)	**3** ^a^	**43** ^a^	**21** ^a^	**803** ^a^	**0.1250** ^a^
	III	4	54	20	792	0.1667
	IV	4	38	20	808	0.1667

**Note:**

a The performance of each indicator at the identified critical stage is marked by bold typeface.

On the other hand, the significant increase in the BCTI score identified the critical state as stage IIB for COAD ( $P = 0.0346$), stage IIIB for LUAD ( $P = 0.0006$), and stage II for THCA ( $P = 0.0302$) (Fig. 3A). These results were consistent with those reported in previous studies (Liu et al., 2022; Zhong et al., 2023). However, the gray curves derived from the mean expression of differentially expressed genes (DEGs) and the genes involved in the networks demonstrated that they cannot signal such critical transitions (more computational details are provided in Note S7 and Fig. S9). Moreover, we also conducted comparative experiments to evaluate the performance of BCTI on the real datasets against other representative approaches, including the edge-based relative entropy (ERE), DNB, and DIND methods (Chen et al., 2012; Peng et al., 2022; Hong et al., 2024) (Figs. 3B–3D and Table S7). The results suggested that compared to other methods, the critical points inferred by BCTI exhibited higher statistical significance (lower $P$-values) and a closer correspondence to clinical observations. Specifically, for COAD, cancer metastasis has not occurred at the identified critical state (stage IIB); however, by stage III, tumor cells have already reached the nearby lymph nodes (Amin et al., 2017). For LUAD, cancer has not metastasized at the identified critical state (stage IIIB), whereas it has already spread to distant tissues or organs *via* the bloodstream at stage IV (Chiang & Massagué, 2008). For THCA, cancer metastasis is absent at the identified critical state (stage II); however, by stage III, cancer has spread to regional lymph nodes (Shaha, 2007). Whereas the ERE, DNB, and DIND methods failed to capture such statistically and biologically meaningful critical points.

To validate the identified critical state, we performed Kaplan–Meier (log-rank) survival analysis for samples separately from before and after the identified critical stage. As depicted in Fig. 3E, the survival expectancy is much higher before the identified critical state than after it, with significant $P$ values observed for COAD, LUAD, and THCA ( $P < 0.0001$, $P = 0.0019$, and $P < 0.0001$, respectively). Moreover, the prognosis analysis also supports the computational results based on BCTI. For example, compared with the prognosis analysis based on other stage divisions, the difference in survival expectancy before and after the identified critical state of THCA by BCTI (stage II) is much more significant ( $P < 0.0001$) (see Fig. S10 for details of the prognosis analysis). These results highlight that patients diagnosed before the identified critical state by BCTI have significantly better prognoses than those diagnosed after the critical stage. More detailed information regarding the survival analysis of tumors can be found in Note S8 and Figs. S11, S12. Taken together, the BCTI score can identify early warning signals for critical states associated with survival expectancy.

In addition, for THCA, we performed Gene Ontology (GO) functional enrichment analysis on differentially expressed genes at each stage. It was found that some regulatory and target genes (such as *IL1A* and *TNF*) (Figs. 3F), although not included in the gold standard, were simultaneously significantly enriched in the same tumor-associated GO terms (such as negative regulation of apoptotic signaling pathway (GO:2001234)) (Figs. 3G and Fig. S13), suggesting the potential biological significance of the regulatory relationships inferred by BCTI (see Tables S8 and S9 for more details). Indeed, in the early stage of THCA, *IL1A* may induce the expression of *TNF*, which could trigger further cascades of inflammatory factors (Guarino et al., 2010). This process might promote tumor cell proliferation and enhance their anti-apoptotic capabilities. Moreover, *IRAK1* and *MYD88* were simultaneously significantly enriched in the regulation of I-kappaB kinase/NF-kappaB signaling (GO:0043122) (Fig. 3F and Table S8). Biologically, chronic inflammation-driven *IRAK1* activity may upregulate *MYD88* expression through pathways such as NF-kappaB, thereby amplifying inflammatory cascades and promoting tumor progression (Jain, Kaczanowska & Davila, 2014). All the results revealed insights into the microscopic change of the underlying signaling transition mechanism, demonstrating BCTI’s capability to offer new perspectives for understanding cancer progression mechanisms and developing precision medicine strategies.

### Leveraging BCTI on single-cell datasets to uncover gene regulatory mechanisms in lung development

**Figure 4:**
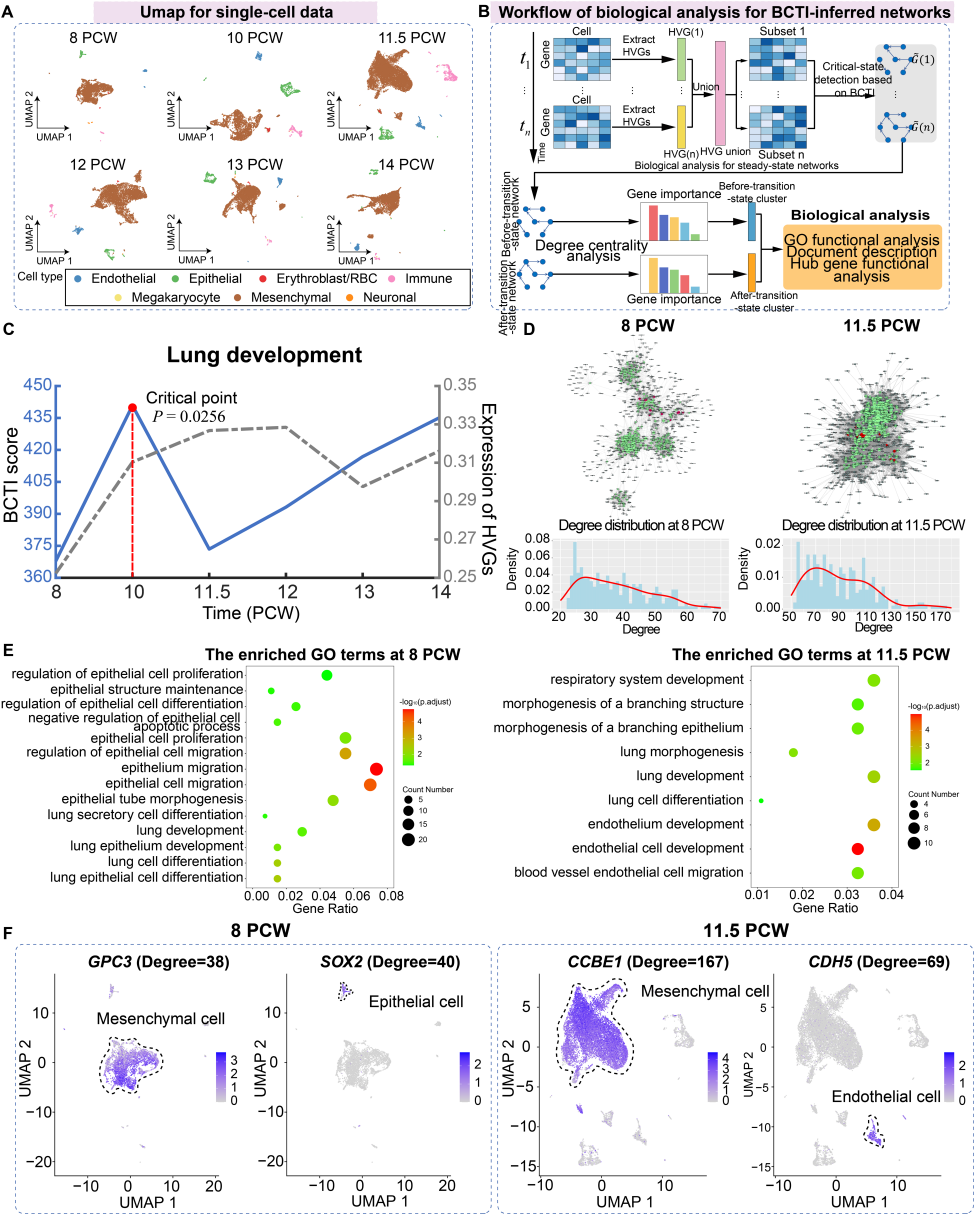
Identifying the critical state and performing biological analysis for lung development. (A) UMAP dimensionality reduction plot of single cells across different time points, with cell-type annotations provided by the original study. (B) Schematic illustration showing the workflow of biological analysis for BCTI-inferred networks. (C) The performance comparison of BCTI and mean expression of HVGs in identifying the critical states. (D) Comparison between the key hub gene networks (top 300 by degree, shown in green) before and after the critical state (8 PCW and 11.5 PCW, respectively). Red nodes denote key hub genes associated with lung development, while gray nodes represent neighboring genes of these hubs. Below panel: degree distribution of key hub genes, indicating increased connectivity between genes after the critical state. (E) GO functional enrichment analysis showed that key hub genes were primarily enriched in epithelium-related lung development before the critical state and in endothelium-related lung development after the critical state. (F) Expression patterns of key hub genes associated with epithelium-related lung development before the critical state and endothelium-related lung development after the critical state.

For the case study of BCTI on human embryonic lung development data, we utilized single-cell data from the pseudoglandular period at 8, 10, 11.5, 12, 13, and 14 post-conception week (PCW), including a total of 42,982 cells and seven main cell types (including mesenchymal, megakaryocyte, epithelial, endothelial, immune, neuronal, and erythroblast/red blood cells) (Fig. 4A) (Sountoulidis et al., 2023).

First, highly variable genes (HVGs) were identified for each stage separately, and their union was used to construct the gene regulatory network. The BCTI method was then applied to quantitatively analyze the dynamic changes in the network, thereby identifying the critical state during lung development (Fig. 4B). As shown in Fig. 4C, the significant increase in the BCTI score identified the critical state as 10 PCW ( $P = 0.0256$). However, the mean expression of the HVGs failed to provide the effective early-warning signal of critical transition during lung development (the gray curve).

Subsequently, key hub genes, ranked by degree, were selected based on degree centrality analysis in the networks before and after the critical state, and biological functional analysis was performed on these genes to explore their downstream functional impacts caused by major regulatory transitions (Fig. 4B). The results showed that, on one hand, in terms of network structure, compared to the before-transition-state GRN, the after-transition-state GRN exhibited tighter connectivity among key hub genes and a higher degree centrality of these genes (Fig. 4D and Fig. S14). It suggested a more consolidated and cooperative regulatory architecture, where key hub genes played an increasingly central role in network stability and functional coordination. On the other hand, in terms of biological function, key hub genes before the critical state were predominantly enriched in functional modules associated with epithelium-related lung development, such as lung epithelium development (GO:0060428) and lung epithelial cell differentiation (GO:0060487), facilitating lung morphogenesis and functional differentiation (Kimura & Deutsch, 2007) (Fig. 4E). However, after the critical state, key hub genes were primarily enriched in functional modules related to endothelium-related lung development, such as morphogenesis of a branching structure (GO:0001763) and endothelial cell development (GO:0001885), highlighting the growing importance of endothelial signaling in supporting lung alveolarization, angiogenesis, and the establishment of the alveolar-capillary network, which are essential for functional gas exchange (Lignelli et al., 2019) (Fig. 4E). Additionally, the expression patterns of certain key hub genes exhibited cell-type heterogeneity, such as *GPC3* and *SOX2* at 8 PCW and *CCBE1* and *CDH5* at 11.5 PCW, further supporting the findings of the GO functional analysis (Fig. 4F). Indeed, the above results aligned with the previous study showing that foetal breathing movements, which are considered to play important roles in lung development (Nikolić, Sun & Rawlins, 2018), begin at 10-11 PCW. More analytical details of BCTI are provided in Table S10. These findings underscore the ability of BCTI to capture dynamic changes in gene regulatory networks that correspond to critical developmental processes.

## Discussion

Inferring gene regulatory networks and detecting critical states in complex biological systems are crucial for understanding biological processes and facilitating targeted interventions. However, achieving both objectives simultaneously remains challenging when dealing with high-dimensional gene expression data. On one hand, many traditional GRN inference methods primarily focus on reconstructing static networks (Huynh-Thu et al., 2010; Wu et al., 2016; Zhang, Chang & Liu, 2021; Iglesias-Martinez, De Kegel & Kolch, 2021; Zhang et al., 2013; Ruyssinck et al., 2014; Guo et al., 2016), making it difficult to uncover the dynamic network features associated with biological processes such as disease progression or cellular differentiation. On the other hand, existing critical-state detection methods often merely identify early-warning signals based on statistical analysis of gene expression fluctuations (Chen et al., 2012, 2019), lacking deeper insights into causal relationships within the network.

In this study, we propose BCTI, a Bayesian network-based framework, to simultaneously infer GRNs and identify critical states in complex biological systems. This framework addresses the limitations of both GRN reconstruction and critical-state detection methods. By comparing BCTI with other benchmark methods across steady-state datasets, including the DREAM network, the IRMA network, and the SOS DNA repair experimental dataset, we demonstrated the stable and superior performance of BCTI in static GRN inference across diverse biological systems. Additionally, by applying BCTI to the 16-node simulated dataset and four real-world datasets (three TCGA datasets and one lung development scRNA-seq dataset), we qualitatively and quantitatively uncovered key features of dynamic network transitions. This allowed us to successfully identify critical states during the evolution of complex biological systems, providing mechanistic insights into system instability, such as cancer onset. The validity of the identified critical points was supported by their consistency with clinical observations and survival analyses, as well as by comparison with previously reported findings (Peng et al., 2022; Liu et al., 2022; Zhong et al., 2023; Hong et al., 2024). Noteworthily, benchmark experiments on simulated datasets with varying noise levels showed that BCTI maintains robust and reliable performance under different data qualities (Fig. S5). The gene regulatory networks inferred by BCTI exhibit a high degree of consistency with well-established KEGG signaling pathways, indicating that the inferred regulatory relationships are biologically interpretable within known cancer-related signaling pathways. In addition, several regulatory relationships beyond these pathways are supported by existing biological literature, while others are further substantiated by Gene Ontology enrichment analysis, which reveals that genes connected by BCTI-inferred regulatory relationships tend to participate in coherent functional modules. Collectively, these lines of evidence support the biological relevance of the regulatory networks inferred by BCTI.

The proposed method serves as a reliable computational tool with the following advantages. First, BCTI offers the comprehensive functionality of simultaneously inferring GRNs and detecting critical states, a capability rarely achieved by traditional methods. Second, BCTI provides a more interpretable approach for signaling critical transitions in complex biological systems, enabling the identification of dynamic changes in molecular regulatory mechanisms during biological progression. Third, BCTI exhibits strong robustness across datasets with varying noise levels, ensuring the reliability of inference results. Finally, as a model-free computational framework, BCTI does not require a model training process and can be applied to different types of biological data, including bulk and single-cell datasets.

Noteworthily, although BCTI demonstrates robust and superior performance across different biological systems, there remains room for further improvement and extension. For example, in large-scale network inference tasks, computational efficiency could be further improved through parallelization strategies. In addition, the integration of deep learning models such as graph neural networks (GNNs) (Scarselli et al., 2008) holds promise for enhancing the method’s ability to capture nonlinear causal relationships.

## Conclusions

The BCTI method provides a novel approach for detecting critical transitions in complex biological systems based on Bayesian network inference. It not only effectively captures early-warning signals of critical transitions in both simulated and real-world datasets, but also reveals dynamic gene regulatory rewiring, providing mechanistic insights into disease progression and developmental processes. Therefore, BCTI holds great practical potential in systems biology, personalized medicine, and the investigation of key molecular regulatory mechanisms driving critical transitions in biological systems.

## Supplemental Information

10.7717/peerj.20860/supp-1 Supplemental Information 1 Supplementary figures, tables, and methodological details. Additional validation results, data analysis, and interpretation that complement the primary results in the main text.

## Additional Information and Declarations

### DNA Deposition

The following information was supplied regarding the deposition of DNA sequences:

The integrated dataset used for analysis and the DREAM, IRMA, and *E. coli* SOS repair network datasets are available at Zenodo: Tong, Y. (2025). Datasets for "BCTI: A Bayesian network-based method for revealing critical transitions in complex biological systems" (v1.0) [Data set]. Zenodo. https://doi.org/10.5281/zenodo.17925561.

The TCGA gene expression datasets (COAD, LUAD, and THCA) are available at the UCSC Xena platform.

The human embryonic lung development single-cell RNA-seq dataset is available at Gene Expression Omnibus (GEO): GSE215895.

All source code and analysis scripts used in this study are available at Zenodo: YuyanTong. (2025). YuyanTong/BCTI: Initial release of BCTI method (v1.1.1). Zenodo. https://doi.org/10.5281/zenodo.15867211.

### Data Availability

The following information was supplied regarding data availability:

The integrated dataset used for analysis and the DREAM, IRMA, and *E. coli* SOS repair network datasets are available at Zenodo: Tong, Y. (2025). Datasets for "BCTI: A Bayesian network-based method for revealing critical transitions in complex biological systems" (v1.0) [Data set]. Zenodo. https://doi.org/10.5281/zenodo.17925561.

The TCGA gene expression datasets (COAD, LUAD, and THCA) are available at the UCSC Xena platform.

The human embryonic lung development single-cell RNA-seq dataset is available at Gene Expression Omnibus (GEO): GSE215895.

All source code and analysis scripts used in this study are available at Zenodo: YuyanTong. (2025). YuyanTong/BCTI: Initial release of BCTI method (v1.1.1). Zenodo. https://doi.org/10.5281/zenodo.15867211.
