# Supplementary material for: BCTI: a Bayesian network-based method for revealing critical transitions in complex biological systems
Source: PeerJ. 2026 Feb 13;14:e20860. doi: 10.7717/peerj.20860 (PMC12908575; doi:10.7717/peerj.20860)
Supplement: Supplemental Information 1 — Additional validation results, data analysis, and interpretation that complement the primary results in the main text. [file peerj-14-20860-s001.docx]

**Supplementary Information of**

**“BCTI: A Bayesian network-based method for revealing critical transitions in complex biological systems”**

Yuyan Tong^1#^, Renhao Hong^1#^, Na Yang^1^, Pei Chen^1^, Hao Peng^1^, Hui Tang^2^*, Rui Liu^1^*

^1^ School of Mathematics, South China University of Technology, Guangzhou 510640, China.

^2^ School of Mathematics, Foshan University, Foshan 528000, China.

*Correspondence: Hui Tang, tanghui@fosu.edu.cn; Rui Liu, scliurui@scut.edu.cn

^#^ These authors contributed equally.

Content

[**Section S1. The supplementary tables** 3](#_Toc189947844)

[Table S1. Genes used for reconstructing gene regulatory networks in each dataset 3](#_Toc189947845)

[Table S2. The number of samples at each stage in different TCGA datasets 4](#_Toc189947846)

[Table S3. Comparison of BCTI’s network inference performance on the DREAM data with other methods 4](#_Toc189947847)

[Table S4. Comparison of BCTI’s network inference performance on the IRMA data with other methods 4](#_Toc189947848)

[Table S5. Comparison of BCTI’s network inference performance on the SOS data with other methods 5](#_Toc189947849)

[Table S6. Network inference performance of BCTI in the numerical simulation 5](#_Toc189947850)

[Table S7. Comparison of the performance among different critical-state detection methods 5](#_Toc189947851)

[Table S8. GO enrichment analysis for the pathway genes in THCA at different stages 6](#_Toc189947852)

[Table S8.1. GO enrichment analysis for the pathway genes in THCA at stage I 6](#_Toc189947853)

[Table S8.2. GO enrichment analysis for the pathway genes in THCA at stage III 6](#_Toc189947854)

[Table S9. Functional analysis for some inferred regulatory relationships in THCA 6](#_Toc189947855)

[Table S10. GO enrichment analysis for the key hub genes in lung development 7](#_Toc189947856)

[Table S10.1. GO enrichment analysis for the key hub genes in lung development at 8 PCW 7](#_Toc189947857)

[Table S10.2. GO enrichment analysis for the key hub genes in lung development at 11.5 PCW 8](#_Toc189947858)

[**Section S2. The supplementary figures** 9](#_Toc189947859)

[Fig. S1. Three states during disease progression 9](#_Toc189947860)

[Fig. S2. An illustration of the “after-transition state” 10](#_Toc189947861)

[Fig. S3. The fluctuation of certain genes during the entire disease progression 11](#_Toc189947862)

[Fig. S4. The gold standard 16-node network in the numerical simulation 11](#_Toc189947863)

[Fig. S5. Comparison of the performance of BCTI under different noise strengths in numerical simulation with other methods 12](#_Toc189947864)

[Fig. S6. Dynamic evolution of the reconstructed network across all stages in COAD 13](#_Toc189947865)

[Fig. S7. Dynamic evolution of the reconstructed network across all stages in LUAD 14](#_Toc189947866)

[Fig. S8. Dynamic evolution of the reconstructed network across all stages in THCA 14](#_Toc189947867)

[Fig. S9. The performance comparison of BCTI and gene expression in identifying the critical states 15](#_Toc189947868)

[Fig. S10. Comparison of the prognosis results based on the identified critical stages by BCTI and other stages 16](#_Toc189947869)

[Fig. S11. A schematic illustration for validating the identified critical state 17](#_Toc189947870)

[Fig. S12. Validating the identified critical states of THCA 18](#_Toc189947871)

[Fig. S13. BCTI uncovered the change of signaling mechanism in the MAPK signaling pathway of THCA 19](#_Toc189947872)

[Fig. S14. Dynamic changes of key hub gene networks before and after the critical transition 20](#_Toc189947873)

[**Section S3. The Supplementary Notes** 21](#_Toc189947874)

[Supplementary Note S1. Dynamic network biomarker (DNB) theory 21](#_Toc189947875)

[Supplementary Note S2. Simplifying mutual information calculation with covariance 21](#_Toc189947876)

[Supplementary Note S3. Explanation for the relationship between the $H$ score and tipping point 22](#_Toc189947877)

[Supplementary Note S4. One-sample *t*-test 26](#_Toc189947878)

[Supplementary Note S5. Details for the data and benchmark methods description 26](#_Toc189947879)

[Supplementary Note S6. Details of numerical simulation 27](#_Toc189947880)

[Supplementary Note S7. Details for the expression calculation of DEGs 30](#_Toc189947881)

[Supplementary Note S8. Verification for the identified critical state 31](#_Toc189947882)

[**Supplementary references** 31](#_Toc189947883)

# Section S1. The supplementary tables

## Table S1. Genes used for reconstructing gene regulatory networks in each dataset

| Dataset | DREAM | IRMA | 16-node simulated network | SOS DNA repair network | COAD | LUAD | THCA |
| --- | --- | --- | --- | --- | --- | --- | --- |
| Number of genes (nodes) | 10 | 5 | 16 | 9 | 15 | 15 | 30 |
| Number of edges | 15 | 6 | 30 | 24 | 15 | 12 | 24 |
| Pathway |  |  |  |  | PI3K-Akt signaling pathway | NF-kappa B signaling pathway | MAPK signaling pathway |
| Gene (Node) names | G1  G2  G3  G4  G5  G6  G7  G8  G9  G10 | *CBF1*  *GAL4*  *SWI5*  *GAL80*  *ASH1* | Node 1  Node 2  Node 3  Node 4  Node 5  Node 6  Node 7  Node 8  Node 9  Node 10  Node 11  Node 12  Node 13  Node 14  Node 15  Node 16 | *lexA*  *dinI*  *umuDC*  *recA*  *ssb*  *recF*  *rpoS*  *rpoH*  *rpoD* | *EGF*  *EGFR*  *GRB2*  *SOS1*  *HRAS*  *RAF1*  *MAP2K1*  *IRS1*  *PIK3CA*  *AKT1*  *MDM2*  *PPP2R1A*  *TCL1A*  *MAPK1*  *TP53* | *BIRC2*  *CD14*  *CHUK*  *IKBKB*  *IL1B*  *IL1R1*  *IRAK1*  *MAP3K14*  *NFKB2*  *NFKBIA*  *RIPK1*  *TICAM2*  *TIRAP*  *TLR4*  *TRAF6* | *ARAF*  *BRAF*  *CASP3*  *CHUK*  *CSF1*  *CSF1R*  *HRAS*  *IL1A*  *IL1R1*  *IRAK1*  *MAP2K5*  *MAP3K2*  *MAP3K5*  *MAP3K7*  *MAP4K2*  *MAPK7*  *MYD88*  *NLK*  *NR4A1*  *PAK1*  *PRKACA*  *RAP1A*  *SOS1*  *STK4*  *TAB1*  *TNF*  *TNFRSF1A*  *TRADD*  *TRAF2*  *TRAF6* |

## Table S2. The number of samples at each stage in different TCGA datasets

| Types of cancer | TA samples | Stage I | | Stage II | | Stage III | | Stage IV |
| --- | --- | --- | --- | --- | --- | --- | --- | --- |
|  |  | Stage IA | Stage IB | Stage IIA | Stage IIB | Stage IIIA | Stage IIIB |  |
| COAD | 41 | 75 | | 165 | 39 | 100 | | 64 |
| LUAD | 59 | 274 | | 51 | 70 | 73 | 11 | 26 |
| THCA | 58 | 281 | | 52 | | 12 | | 55 |

TA samples: tumor-adjacent samples

## Table S3. Comparison of BCTI’s network inference performance on the DREAM data with other methods

| Indices  Methods | TP | FN | FP | TN | Precision | TPR | FPR | Specificity | Accuracy | Error |
| --- | --- | --- | --- | --- | --- | --- | --- | --- | --- | --- |
| **BCTI** | 6 | 9 | 4 | 71 | 0.6000 | 0.4000 | 0.0533 | 0.9467 | 0.8556 | 0.1444 |
| GENMS | 3 | 12 | 5 | 70 | 0.3750 | 0.2000 | 0.0667 | 0.9333 | 0.8111 | 0.1889 |
| PLSNET | 2 | 13 | 13 | 62 | 0.1333 | 0.1333 | 0.1733 | 0.8267 | 0.7111 | 0.2889 |
| NIMEFI | 1 | 14 | 12 | 63 | 0.0769 | 0.0667 | 0.1600 | 0.8400 | 0.7111 | 0.2889 |
| GENIE3_RF | 5 | 10 | 10 | 65 | 0.3333 | 0.3333 | 0.1333 | 0.8667 | 0.7778 | 0.2222 |
| GENIE3_ET | 7 | 8 | 17 | 58 | 0.2917 | 0.4667 | 0.2267 | 0.7733 | 0.7222 | 0.2778 |
| GNIPLR | 2 | 13 | 11 | 64 | 0.1538 | 0.1333 | 0.1467 | 0.8533 | 0.7333 | 0.2667 |
| NARROMI | 5 | 10 | 14 | 61 | 0.2632 | 0.3333 | 0.1867 | 0.8133 | 0.7333 | 0.2667 |

## Table S4. Comparison of BCTI’s network inference performance on the IRMA data with other methods

| Indices  Methods | TP | FN | FP | TN | Precision | TPR | FPR | Specificity | Accuracy | Error |
| --- | --- | --- | --- | --- | --- | --- | --- | --- | --- | --- |
| **BCTI** | 4 | 2 | 1 | 13 | 0.8000 | 0.6667 | 0.0714 | 0.9286 | 0.8500 | 0.1500 |
| GENMS | 5 | 1 | 7 | 7 | 0.4167 | 0.8333 | 0.5000 | 0.5000 | 0.6000 | 0.4000 |
| PLSNET | 2 | 4 | 5 | 9 | 0.2857 | 0.3333 | 0.3571 | 0.6429 | 0.5500 | 0.4500 |
| NIMEFI | 1 | 5 | 0 | 14 | 1.0000 | 0.1667 | 0.0000 | 1.0000 | 0.7500 | 0.2500 |
| GENIE3_RF | 4 | 2 | 6 | 8 | 0.4000 | 0.6667 | 0.4286 | 0.5714 | 0.6000 | 0.4000 |
| GENIE3_ET | 3 | 3 | 5 | 9 | 0.3750 | 0.5000 | 0.3571 | 0.6429 | 0.6000 | 0.4000 |
| GNIPLR | 5 | 1 | 4 | 10 | 0.5556 | 0.8333 | 0.2857 | 0.7143 | 0.7500 | 0.2500 |
| NARROMI | 3 | 3 | 2 | 12 | 0.6000 | 0.5000 | 0.1429 | 0.8571 | 0.7500 | 0.2500 |

## Table S5. Comparison of BCTI’s network inference performance on the SOS data with other methods

| Indices  Method | TP | FP | FN | TN | Precision | TPR | FPR | Specificity | Accuracy | Error |
| --- | --- | --- | --- | --- | --- | --- | --- | --- | --- | --- |
| **BCTI** | 14 | 10 | 10 | 38 | 0.5833 | 0.5833 | 0.2083 | 0.7917 | 0.7222 | 0.2778 |
| CLR | 5 | 13 | 19 | 35 | 0.2778 | 0.2083 | 0.2708 | 0.7292 | 0.5556 | 0.4444 |
| ARACNE | 2 | 8 | 22 | 40 | 0.2000 | 0.0833 | 0.1667 | 0.8333 | 0.5833 | 0.4167 |
| GENIE3-RF-all | 8 | 10 | 16 | 38 | 0.4444 | 0.3333 | 0.2083 | 0.7917 | 0.6389 | 0.3611 |
| GENIE3-ET-all | 10 | 5 | 14 | 43 | 0.6667 | 0.4167 | 0.1042 | 0.8958 | 0.7361 | 0.2639 |
| GENIE3-RF-sqrt | 7 | 8 | 17 | 40 | 0.4667 | 0.2917 | 0.1667 | 0.8333 | 0.6528 | 0.3472 |
| GENIE3-ET-sqrt | 9 | 7 | 15 | 41 | 0.5625 | 0.3750 | 0.1458 | 0.8542 | 0.6944 | 0.3056 |
| GENIMS | 8 | 13 | 16 | 35 | 0.3810 | 0.3333 | 0.2708 | 0.7292 | 0.5972 | 0.4028 |
| NIMEFI | 8 | 6 | 16 | 42 | 0.5714 | 0.3333 | 0.1250 | 0.8750 | 0.6944 | 0.3056 |
| PLSNET | 6 | 6 | 18 | 42 | 0.5000 | 0.2500 | 0.1250 | 0.8750 | 0.6667 | 0.3333 |
| NARROMI | 7 | 13 | 17 | 35 | 0.3500 | 0.2917 | 0.2708 | 0.7292 | 0.5833 | 0.4167 |
| GNIPLR | 14 | 3 | 10 | 45 | 0.8235 | 0.5833 | 0.0625 | 0.9375 | 0.8194 | 0.1806 |

## Table S6. Network inference performance of BCTI in the numerical simulation

| Indices  Parameters | TP | FN | FP | TN | Precision | TPR | FPR | Specificity | Accuracy | Error |
| --- | --- | --- | --- | --- | --- | --- | --- | --- | --- | --- |
| -0.3 | 11 | 19 | 22 | 188 | 0.3333 | 0.3667 | 0.1048 | 0.8952 | 0.8292 | 0.1708 |
| -0.2 | 12 | 18 | 29 | 181 | 0.2927 | 0.4000 | 0.1381 | 0.8619 | 0.8042 | 0.1958 |
| -0.1 | 11 | 19 | 25 | 185 | 0.3056 | 0.3667 | 0.1190 | 0.8810 | 0.8167 | 0.1833 |
| -0.001 | 6 | 24 | 12 | 198 | 0.3333 | 0.2000 | 0.0571 | 0.9429 | 0.8500 | 0.1500 |
| 0.1 | 9 | 21 | 29 | 181 | 0.2368 | 0.3000 | 0.1381 | 0.8619 | 0.7917 | 0.2083 |
| 0.2 | 10 | 20 | 21 | 189 | 0.3226 | 0.3333 | 0.1000 | 0.9000 | 0.8292 | 0.1708 |
| 0.3 | 11 | 19 | 29 | 181 | 0.3333 | 0.3667 | 0.1048 | 0.8952 | 0.8292 | 0.1708 |

## Table S7. Comparison of the performance among different critical-state detection methods

| Datasets  Method | COAD | LUAD | THCA |
| --- | --- | --- | --- |
| BCTI | Stage IIB (P=0.03466) | Stage IIIB (P=0.0006) | Stage II (P=0.0302) |
| ERE | None | Stage IIIB (P=0.0001) | None |
| DNB | None | Stage IV (P=0.0106) | None |
| DIND | None | Stage IIIB (P=0.00013) | Stage II (P=0.026) |

## Table S8. GO enrichment analysis for the pathway genes in THCA at different stages

### Table S8.1. GO enrichment analysis for the pathway genes in THCA at stage I

| Enriched Gene Ontology (GO) terms | Gene Ratio | Enriched p-adjust | Involved pathway genes |
| --- | --- | --- | --- |
| Regulation of cell-cell adhesion (GO:0022407) | 0.0285 | 0.0002 | *TRAF6*/*CASP3*/*IRAK1*/*MAPK7*/*IL1A*/*TNF*/*MAP2K5* |
| Negative regulation of immune system process (GO:0043123) | 0.0129 | 0.0003 | *TRAF6*/*TNFRSF1A*/*IRAK1*/*MYD88*/*CHUK*/*TNF* |
| Regulation of I-kappaB kinase/NF-kappaB signaling (GO:0043122) | 0.0166 | 0.0004 | *TRAF6*/*TNFRSF1A*/*IRAK1*/*MYD88*/*CHUK*/*TNF* |
| Positive regulation of NF-kappaB transcription factor activity (GO:0051092) | 0.0106 | 0.0007 | *TRAF6*/*IRAK1*/*MYD88*/*CHUK*/*TNF* |
| I-kappaB kinase/NF-kappaB signaling (GO:0007249) | 0.0183 | 0.0008 | *TRAF6*/*TNFRSF1A*/*IRAK1*/*MYD88*/*CHUK*/*TNF* |
| Negative regulation of apoptotic signaling pathway (GO:2001234) | 0.0147 | 0.0029 | *MAPK7*/*IL1A/TNF*/*MAP2K5* |
| Cellular response to tumor necrosis factor (GO:0071356) | 0.0150 | 0.0030 | *TRAF6*/*TNFRSF1A*/*CHUK*/*MAP3K5*/*TNF* |
| Response to tumor necrosis factor (GO:0034612) | 0.0163 | 0.0042 | *TRAF6*/*TNFRSF1A*/*CASP3*/*CHUK*/*MAP3K5*/*TNF* |
| T cell proliferation (GO:0042098) | 0.0125 | 0.0409 | *TRAF6*/*CASP3*/*SOS1*/*IL1A* |

### Table S8.2. GO enrichment analysis for the pathway genes in THCA at stage III

| Enriched Gene Ontology (GO) terms | Gene Ratio | Enriched p-adjust | Involved pathway genes |
| --- | --- | --- | --- |
| Regulation of I-kappaB kinase/NF-kappaB signaling (GO:0043122) | 0.0159 | 0.0083 | *TRAF6*/*TNFRSF1A*/*MYD88*/*IRAK1*/*CHUK*/*TNF*/*MAP3K7*/*TRADD* |
| Positive regulation of I-kappaB kinase/NF-kappaB signaling (GO:0043123) | 0.0120 | 0.0152 | *TRAF6*/*TNFRSF1A*/*MYD88*/*IRAK1*/*CHUK*/*TNF*/*MAP3K7*/*TRADD* |
| I-kappaB kinase/NF-kappaB signaling (GO:0007249) | 0.0174 | 0.0283 | *TRAF6*/*TNFRSF1A*/*MYD88*/*IRAK1*/*CHUK*/*TNF*/*MAP3K7*/*TRADD* |
| Cellular response to tumor necrosis factor (GO:0071356) | 0.0143 | 0.0360 | *TRAF6*/*TNFRSF1A*/*CHUK*/*MAP3K5*/*TNF*/*TRADD* |
| Cell chemotaxis (GO:0060326) | 0.0190 | 0.0361 | *NR4A1*/*CSF1R*/*CSF1* |
| Response to tumor necrosis factor (GO:0034612) | 0.0156 | 0.0435 | *TRAF6*/*TNFRSF1A*/*CASP3*/*CHUK*/*MAP3K5*/*TNF*/*TRADD* |
| Positive regulation of NF-kappaB transcription factor activity (GO:0051092) | 0.0098 | 0.0472 | *TRAF6*/*MYD88*/*IRAK1*/*CHUK*/*TNF*/*MAP3K7*/*TRADD* |

## Table S9. Functional analysis for some inferred regulatory relationships in THCA

| Gene | Inferred regulatory relationship | Type | Location | Family | Relation with tumors |
| --- | --- | --- | --- | --- | --- |
| *SOS1* | *CASP3*$\to$*SOS1* | Target gene | Cytosol | Guanine nucleotide exchange factor | *SOS1* activates *RAS*, and its abnormal expression or mutations can trigger uncontrolled cell growth, leading to tumor development [1]. |
| *TNFRSF1A* | *CASP3*$\to$*TNFRSF1A* | Target gene | Extracellular | TNF receptor | Overexpression or abnormal activation of *TNFRSF1A* may promote tumor formation by driving abnormal cell proliferation and anti-apoptotic signaling [2]. |
| *CHUK* | *CHUK*$\to$*MAP3K5* | Source gene | Nucleus | Protein kinase | *CHUK* abnormalities or mutations may cause sustained NF-κB activation, driving tumor survival, metastasis, and resistance [3]. |
| *MAP3K5* | *CHUK*$\to$*MAP3K5* | Target gene | Cytosol | Protein kinase | Overexpression or abnormal activation of *MAP3K5* may lead to increased cell survival and resistance to apoptosis, which can contribute to cancer development [4]. |
| *IL1A* | *IL1A*$\to$*TNF* | Source gene | Extracellular | Cytokine | Overexpression of *IL1A* can promote a pro-inflammatory tumor microenvironment, supporting tumor growth, invasion, and metastasis [5]. |
| *HRAS* | *HRAS*$\to$*TNFRSF1A* | Source gene | Nucleus | Oncogene | Elevated *HRAS* expression activates the RAS pathway, promoting tumor growth and metastasis [6]. |

## Table S10. GO enrichment analysis for the key hub genes in lung development

### Table S10.1. GO enrichment analysis for the key hub genes in lung development at 8 PCW

| Enriched Gene Ontology (GO) terms | Gene Ratio | Enriched p-adjust | Involved hub genes |
| --- | --- | --- | --- |
| Regulation of epithelial cell proliferation (GO:0050678) | 0.0441 | 0.0457 | *APLNR/SCG2/SOX2/GPC3/HMGB1/KDR/TIE1/DLL4/MEF2C/SERPINF1/SNAI2/FLT1* |
| Epithelial structure maintenance (GO:0010669) | 0.0110 | 0.0423 | *TFF3/CXADR/LDB2* |
| Regulation of epithelial cell differentiation (GO:0030856) | 0.0257 | 0.0355 | *CD24/CLDN5/CDH5/IL1B/GRHL2/MMP9/PRKCH* |
| Negative regulation of epithelial cell apoptotic process (GO:1904036) | 0.0147 | 0.0307 | *CDH5/SCG2/KDR/RAMP2* |
| Epithelial cell proliferation (GO:0050673) | 0.0551 | 0.0138 | *APLNR/CD34/SCG2/SOX2/GPC3/HMGB1/KDR/TIE1/DLL4/MEF2C/SERPINF1/SNAI2/FGL1/FLT1/KIT* |
| Regulation of epithelial cell migration (GO:0010632) | 0.0551 | 0.0006 | *TMSB4X/ANXA3/DCN/PTPRR/EDN1/ANXA1/HMGB1/KDR/DLL4/MEF2C/MMP9/TAC1/CCBE1/HDAC9/SERPINF1* |
| Epithelium migration (GO:0090132) | 0.0735 | <0.0001 | *TMSB4X/CDH5/ANXA3/DCN/PTPRR/SCG2/EDN1/ZEB2/ANXA1/HMGB1/KDR/GRHL2/DLL4/MEF2C/MMP9/TAC1/CCBE1/HDAC9/SERPINF1/KIT* |
| Epithelial cell migration (GO:0010631) | 0.0699 | 0.0001 | *TMSB4X/CDH5/ANXA3/DCN/PTPRR/SCG2/EDN1/ZEB2/ANXA1/HMGB1/KDR/DLL4/MEF2C/MMP9/TAC1/CCBE1/HDAC9/SERPINF1/KIT* |
| Epithelial tube morphogenesis (GO:0060562) | 0.0478 | 0.0086 | *CSF1R/APLNR/EDN1/GPC3/HAND2/SOX17/NKX2-1/KDR/TIE1/GRHL2/DLL4/MEF2C/FMN1* |
| Lung secretory cell differentiation (GO:0061140) | 0.0074 | 0.0407 | *NKX2-1/AGR2* |
| Lung development (GO:0030324) | 0.0294 | 0.0249 | *GPC3/KLF2/EPAS1/HMGB1/NKX2-1/GRHL2/CCBE1/AGR2* |
| Lung epithelium development (GO:0060428) | 0.0147 | 0.0141 | *KLF2/NKX2-1/GRHL2/AGR2* |
| Lung cell differentiation (GO:0060479) | 0.0147 | 0.0042 | *KLF2/NKX2-1/GRHL2/AGR2* |
| Lung epithelial cell differentiation (GO:0060487) | 0.0147 | 0.0036 | *KLF2/NKX2-1/GRHL2/AGR2* |

### Table S10.2. GO enrichment analysis for the key hub genes in lung development at 11.5 PCW

| Enriched Gene Ontology (GO) terms | Gene Ratio | Enriched p-adjust | Involved hub genes |
| --- | --- | --- | --- |
| Respiratory system development (GO:0060541) | 0.0360 | 0.0070 | *CCBE1/GPC3/NFIB/TNF/AGR2/GRHL2/RSPO2/TCF21/HOPX/DNAAF1* |
| Blood vessel endothelial cell migration (GO:0043534) | 0.0324 | 0.0090 | *ANXA1/HDAC9/TNF/MEOX2/HMOX1/PPARG/SLIT2/CDH5/ANGPT1* |
| Morphogenesis of a branching structure (GO:0001763) | 0.0324 | 0.0162 | *GPC3/TNF/PBX1/SLIT2/IL10/GRHL2/EDN1/RSPO2/TCF21* |
| Morphogenesis of a branching epithelium (GO:0061138) | 0.0324 | 0.0110 | *GPC3/TNF/PBX1/SLIT2/IL10/GRHL2/EDN1/RSPO2/TCF21* |
| Endothelium development (GO:0003158) | 0.0360 | 0.0005 | *TNF/IL1B/CDH5/CLDN5/CLDN3/PPP1R16B/PDE4D/PECAM1/CD34/ICAM1* |
| Endothelial cell development (GO:0001885) | 0.0324 | <0.0001 | *TNF/IL1B/CDH5/CLDN5/CLDN3/PPP1R16B/PDE4D/PECAM1/ICAM1* |
| Lung morphogenesis (GO:0060425) | 0.0180 | 0.0064 | *NFIB/TNF/GRHL2/RSPO2/TCF21* |
| Lung development (GO:0030324) | 0.0360 | 0.0030 | *CCBE1/GPC3/NFIB/TNF/AGR2/GRHL2/RSPO2/TCF21/HOPX/DNAAF1* |
| Lung cell differentiation (GO:0060479) | 0.0108 | 0.0264 | *NFIB/AGR2/GRHL2* |

# Section S2. The supplementary figures

## Fig. S1. Three states during disease progression


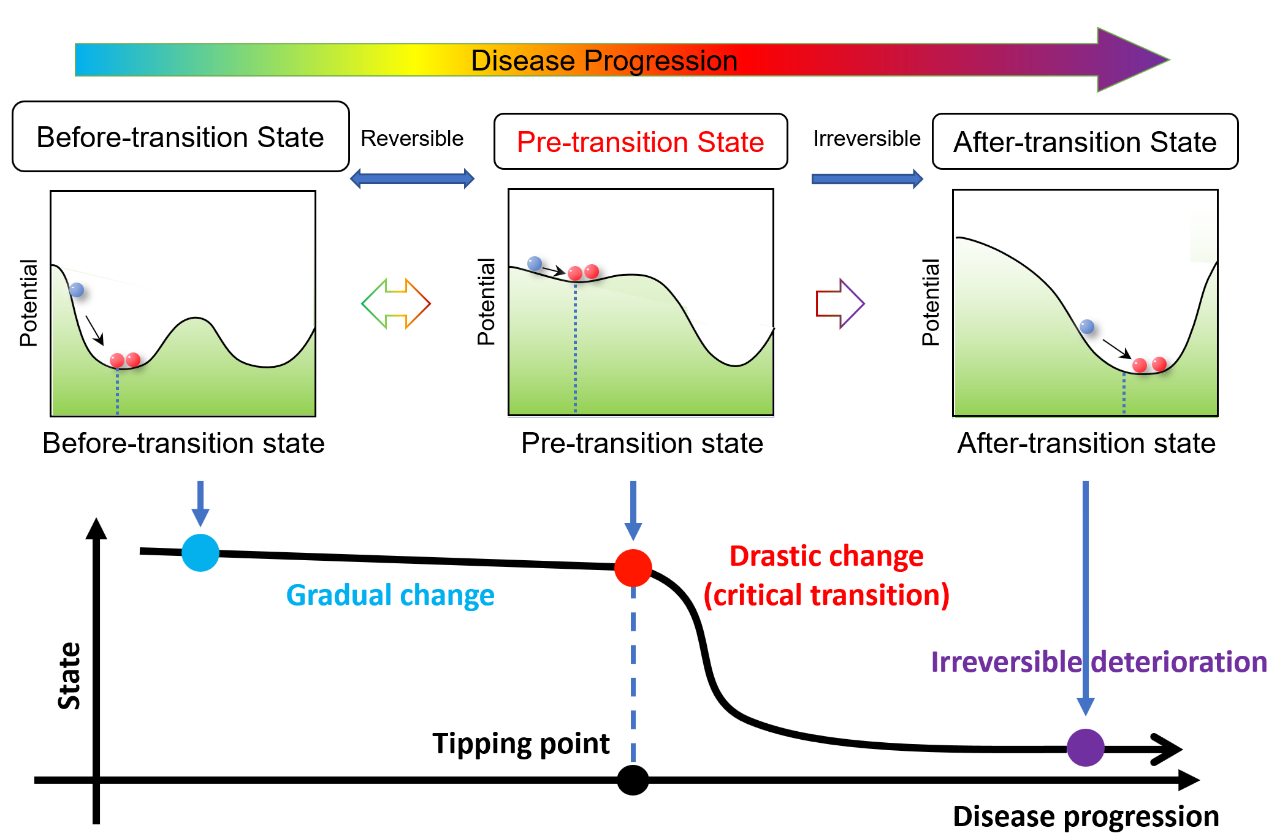


**Figure S1.** **Three states during disease progression.** The before-transition state is a steady or stable state with strong resilience for small perturbations, representing a relatively healthy stage. The pre-transition/critical state is defined as the limit of the before-transition state but with a lower resilience from small perturbations. Such a pre-transition state is the critical stage during the disease progression. When the system is at the pre-transition state, timely and proper medical intervention can bring the system back to the before-transition state. The after-transition state is another stable state with strong resilience, where the system turns into a severe deterioration stage and thus it is generally difficult to return to the before-transition state even by the intensive medical treatment.

## Fig. S2. An illustration of the “after-transition state”


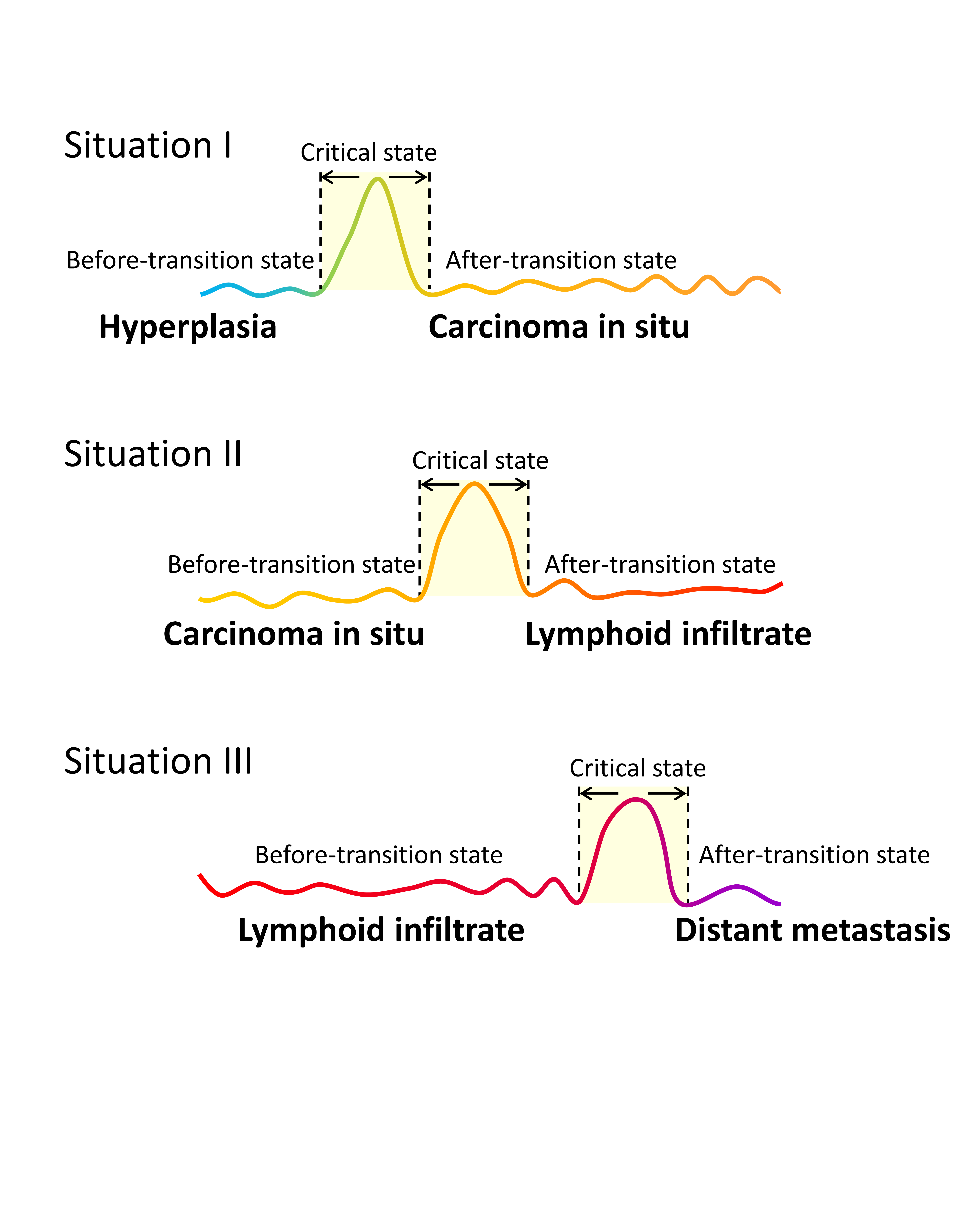


**Figure S2. An illustration of the “after-transition state”.** There may be multiple critical states occurring during disease progression. If the state after the critical state shows drastic deterioration compared to before, we consider it the “after-transition state”. We take cancer as an example. (I) Patients experience a drastic deterioration at the stage of carcinoma in situ compared to the hyperplasia stage. Therefore, the stage of carcinoma in situ is considered as the “after-transition state”. (II) The lymphoid infiltrates are more severe deterioration compared to the stage of carcinoma in situ, and is considered as the “after-transition state”. (III) The stage of distant metastasis is a more severe stage compared to the stage of lymphoid infiltrates, almost incurable, and is considered as the “after-transition state”.

## Fig. S3. The fluctuation of certain genes during the entire disease progression


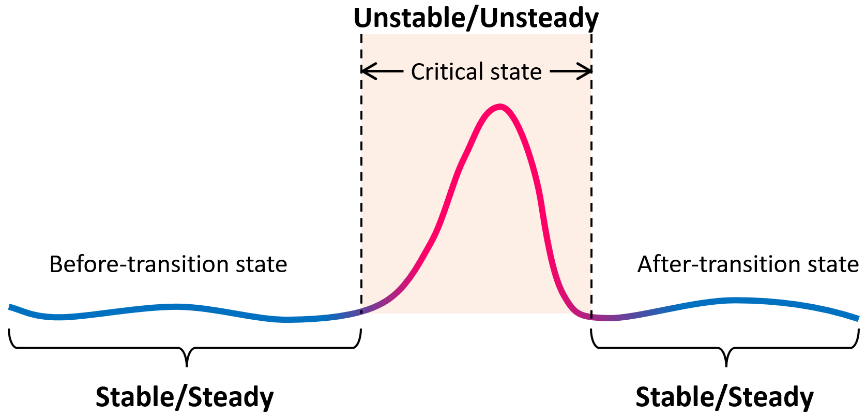


**Figure S3. The fluctuation of certain genes during the entire disease progression.** Specifically, the fluctuation of expression of certain genes (nodes) may exhibit rapid increase in the critical state, while they remain low both in the before-transition and after-transition states.

## Fig. S4. The gold standard 16-node network in the numerical simulation


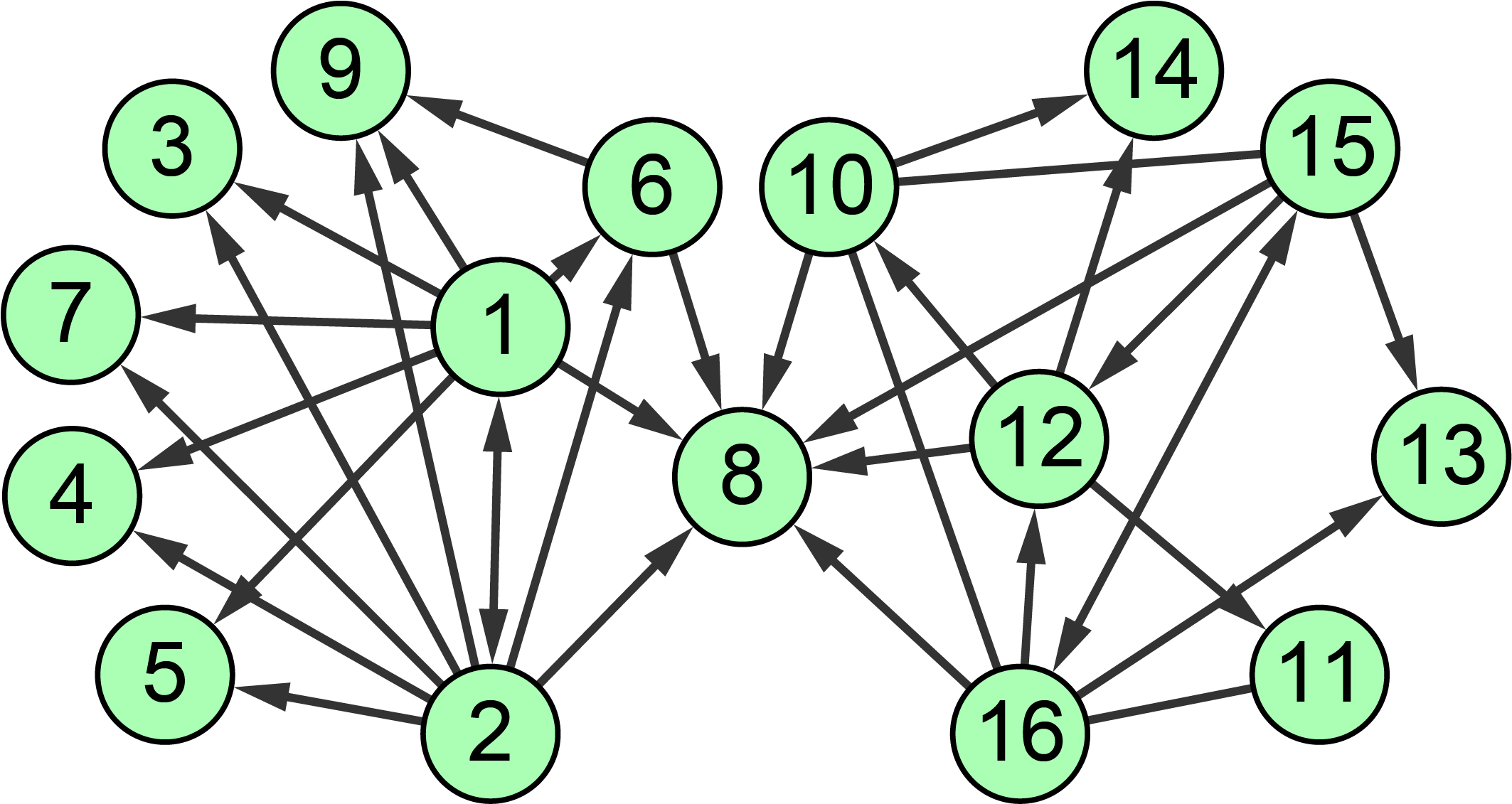


**Figure S4.** **The gold standard 16-node network in the numerical simulation.** In the network, the nodes represent genes, and the edges represent positive or negative regulations among genes. The background differential equation set is shown as Eq. (S22).

## Fig. S5. Comparison of the performance of BCTI under different noise strengths in numerical simulation with other methods


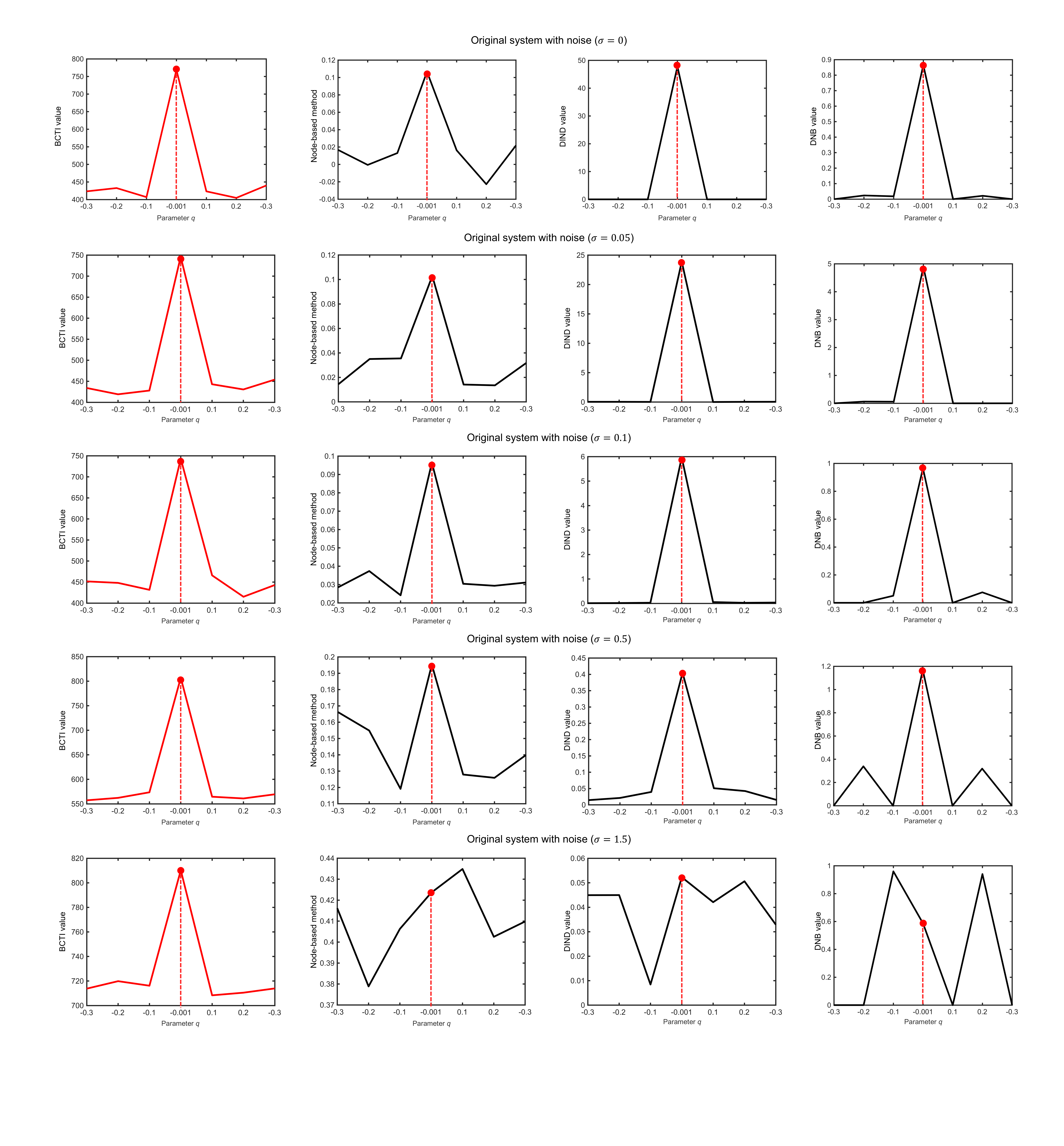


**Figure S5.** **Comparison of the performance of BCTI under different noise strengths in numerical simulation with other methods.** Even though the noise strength increases, BCTI maintains a stable curve trend and provides distinct early-warning signals, validating the robustness of the BCTI method.

## Fig. S6. Dynamic evolution of the reconstructed network across all stages in COAD


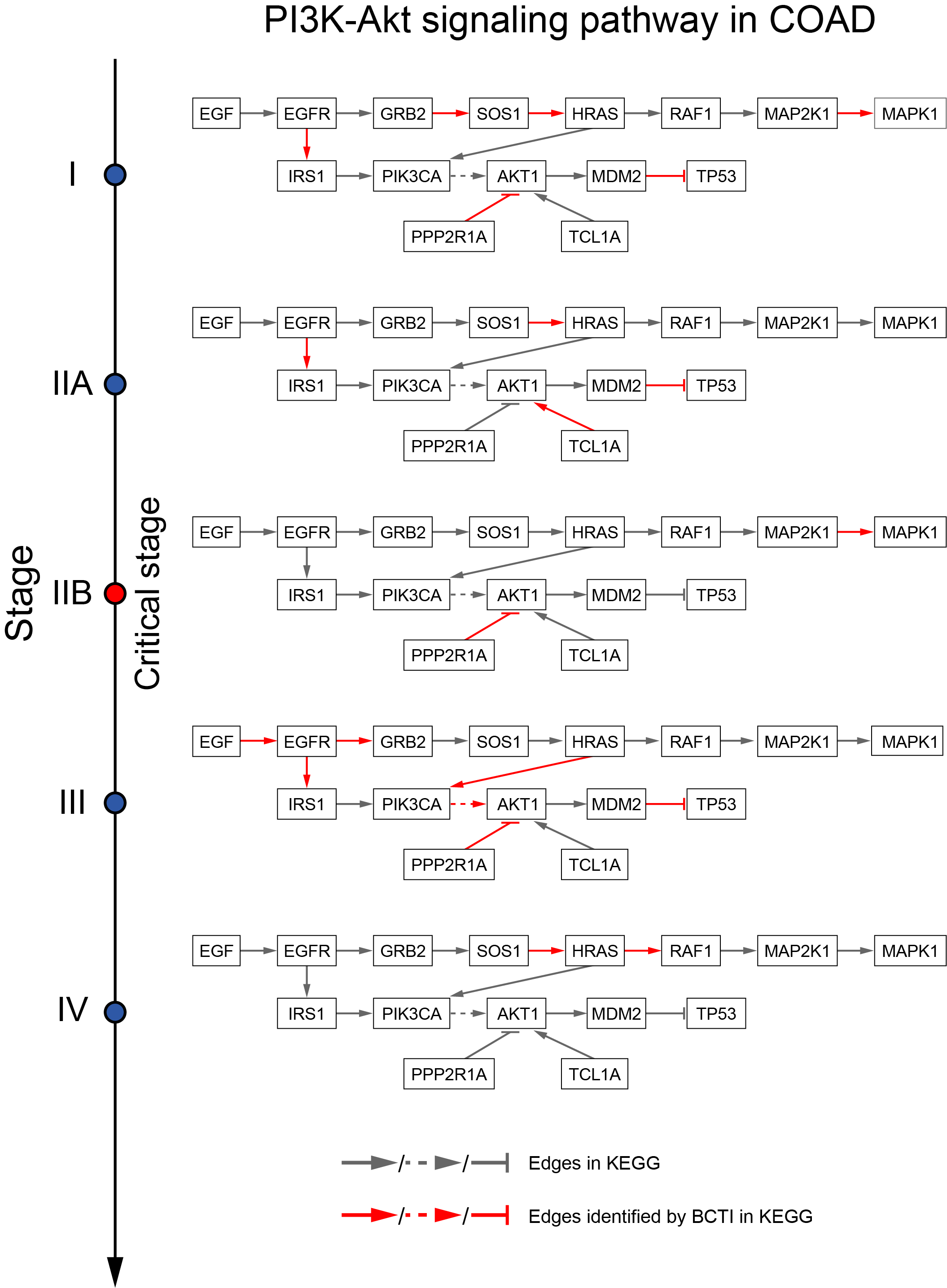


**Figure S6. Dynamic evolution of the reconstructed network across all stages in COAD.** BCTI correctly identified 6, 4, 2, 7, and 2 edges in stages I, IIA, IIB, III, and IV, respectively. This sharp reduction in the number of correctly predicted edges ($TP=2$) at the critical point (stage IIB) indicates increased instability in the system’s state.

## Fig. S7. Dynamic evolution of the reconstructed network across all stages in LUAD


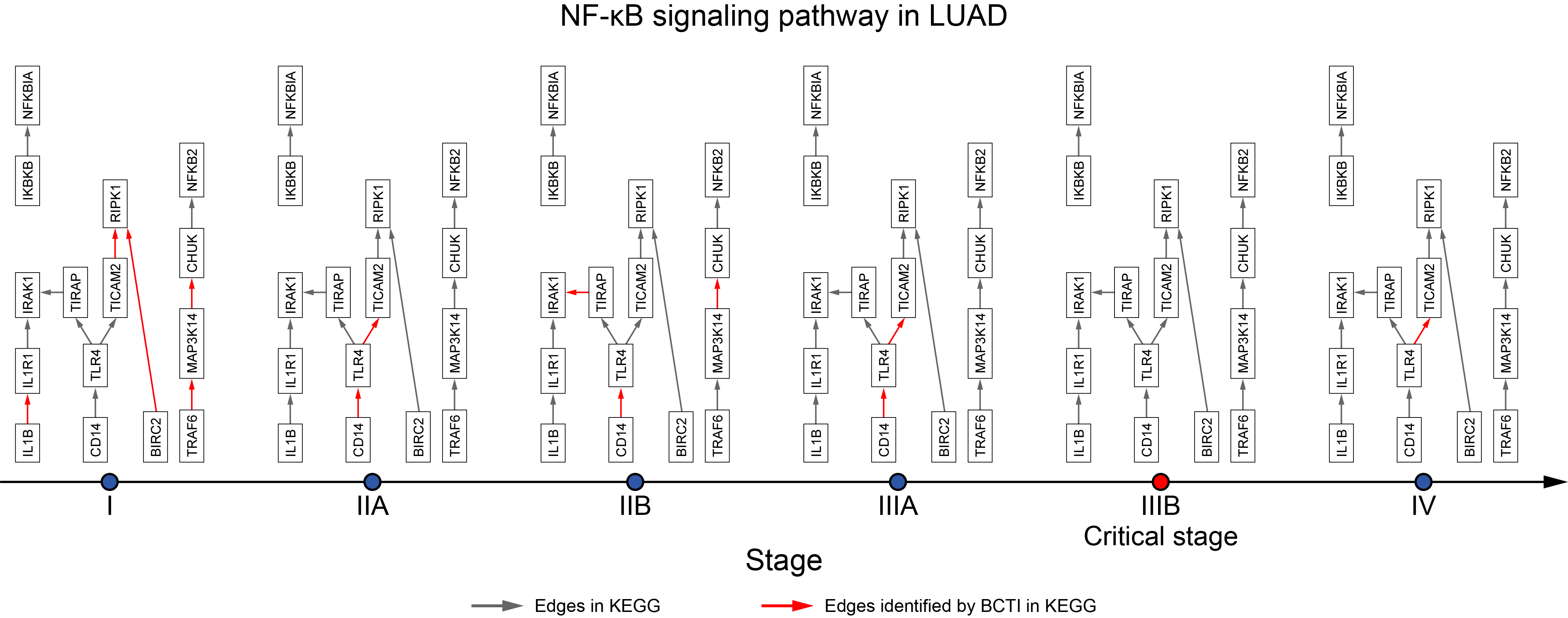


**Figure S7. Dynamic evolution of the reconstructed network across all stages in LUAD.** BCTI correctly identified 5, 2, 3, 2, 0, and 1 edges in stages I, IIA, IIB, IIIA, IIIB, and IV, respectively. The sharp reduction to zero correctly predicted edges ($TP=0$) at the critical point (stage IIIB) indicates increased instability in the system’s state.

## Fig. S8. Dynamic evolution of the reconstructed network across all stages in THCA


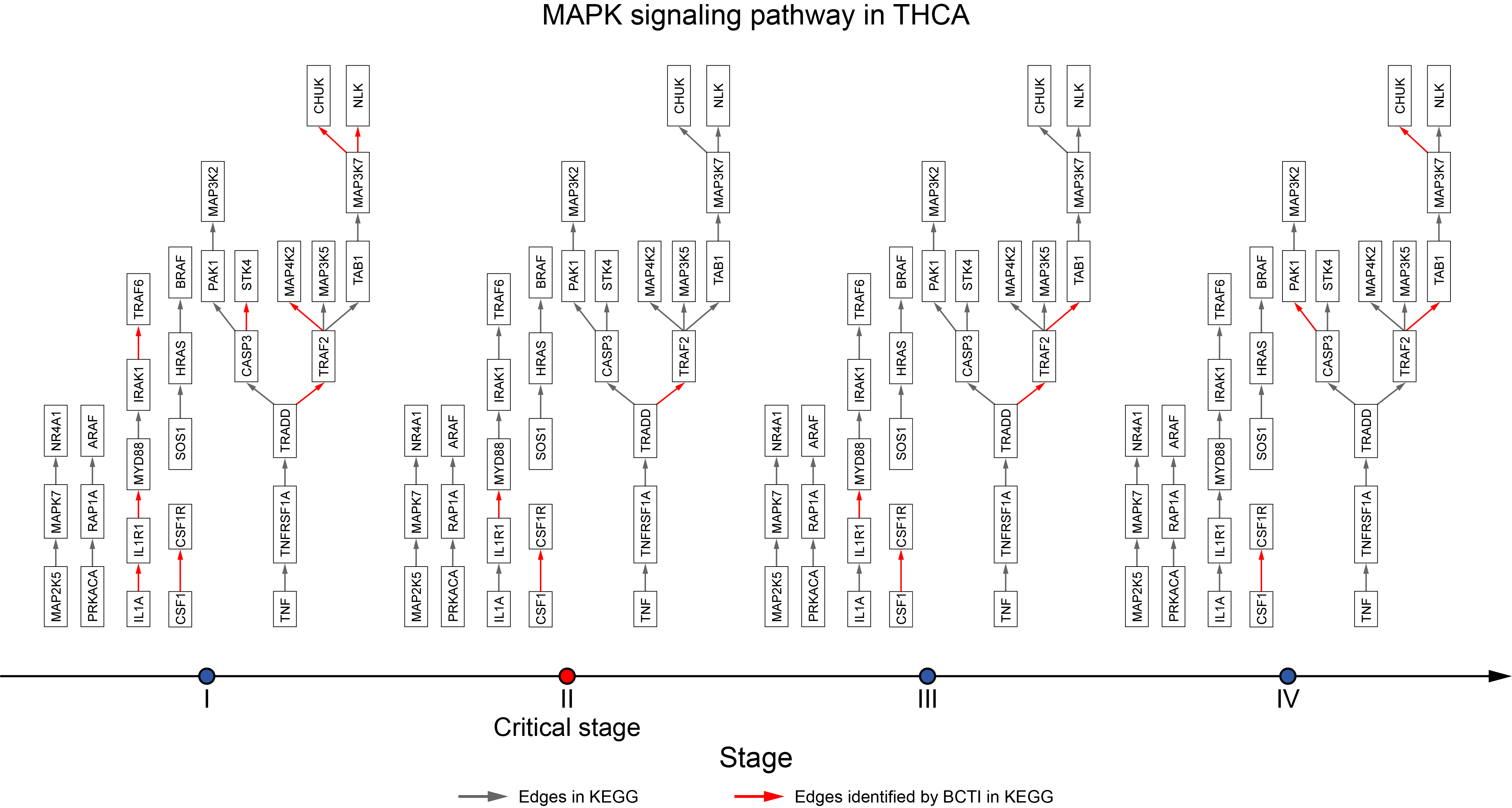


**Figure S8. Dynamic evolution of the reconstructed network across all stages in THCA.** BCTI correctly identified 9, 3, 4, and 4 edges in stages I, II, III, and IV, respectively. This sharp reduction in the number of correctly predicted edges ($TP=3$) at the critical point (stage II) indicates increased instability in the system’s state.

## Fig. S9. The performance comparison of BCTI and gene expression in identifying the critical states


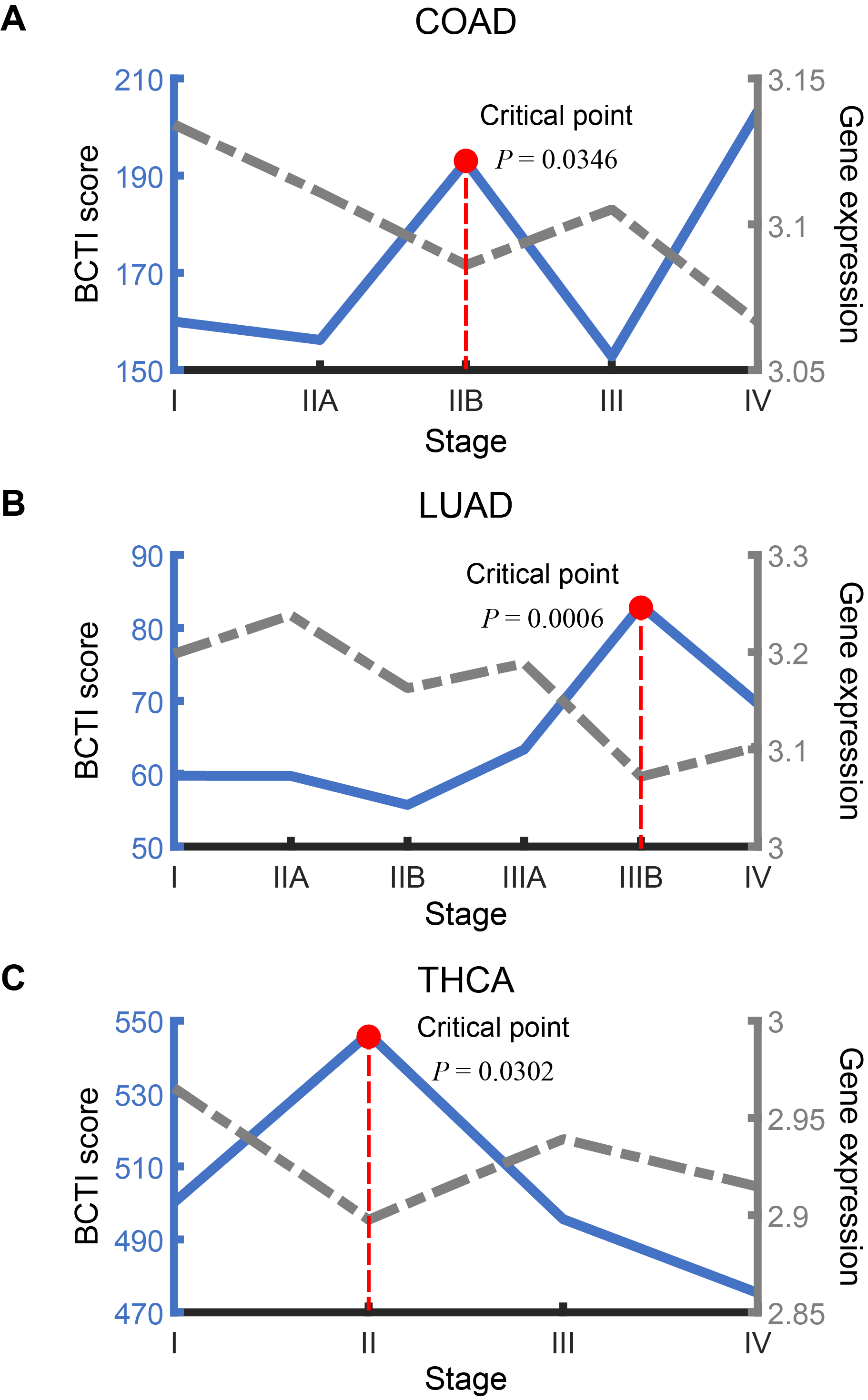


**Figure S9.** **Identifying critical states for tumor deterioration based on different methods.** The performance comparison of BCTI and average expression of the genes comprising the reconstructed networks in identifying the critical states for different tumor datasets: (A) COAD, (B) LUAD, and (C) THCA.

## Fig. S10. Comparison of the prognosis results based on the identified critical stages by BCTI and other stages


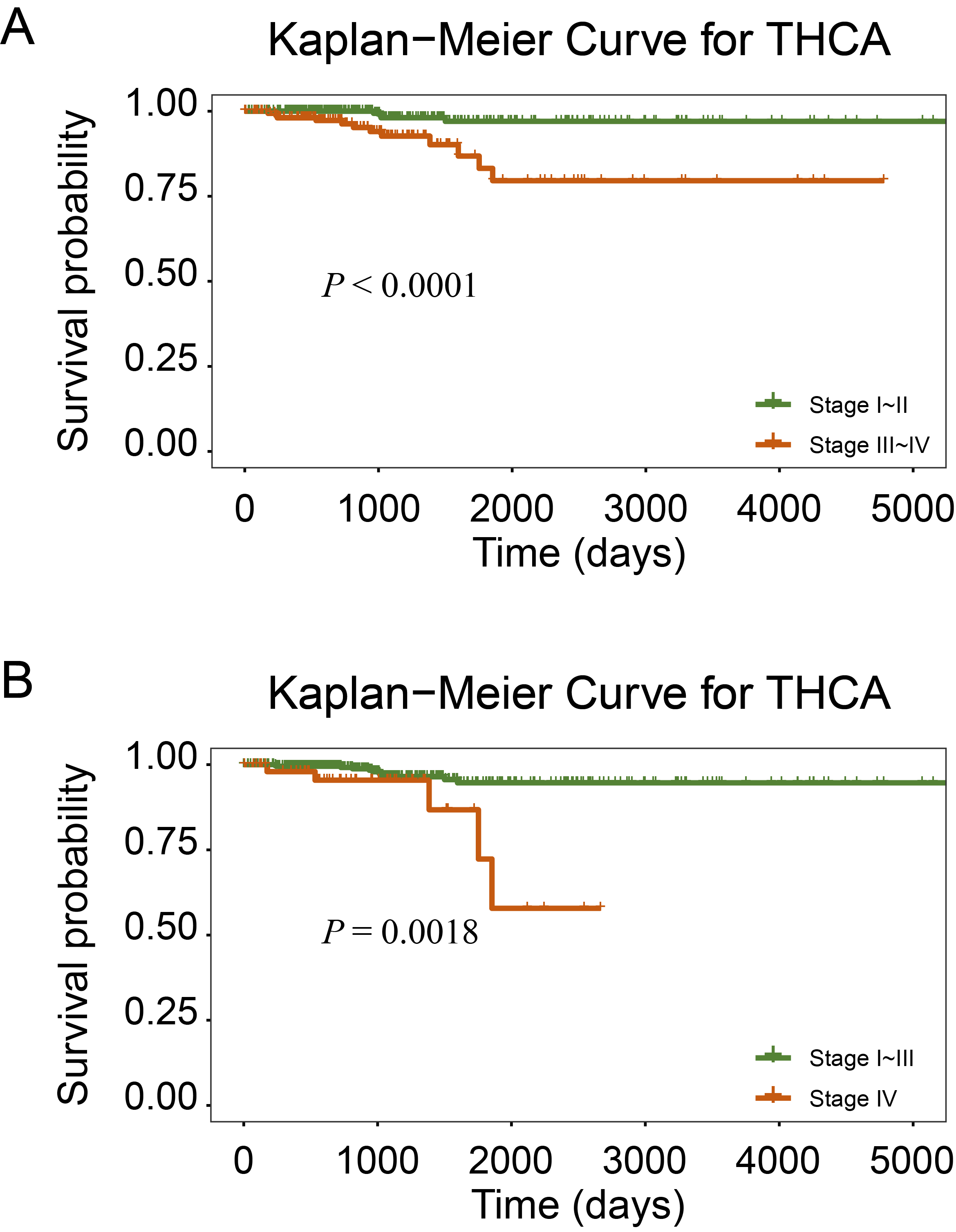


**Figure S10. Comparison of the prognosis results based on the identified critical stages by BCTI and other stages for THCA. (A)** Survival curves before and after the critical stage II (identified by the BCTI method) in THCA patients ($P<0.0001$). **(B)** Survival curves before and after the critical stage III in THCA patients ($P=0.0018$). Notably, there is a more significant difference between the survival curves before and after stage II than stage III, validating that there is a critical transition that leads to different survival time in stage II instead of stage III.

## Fig. S11. A schematic illustration for validating the identified critical state


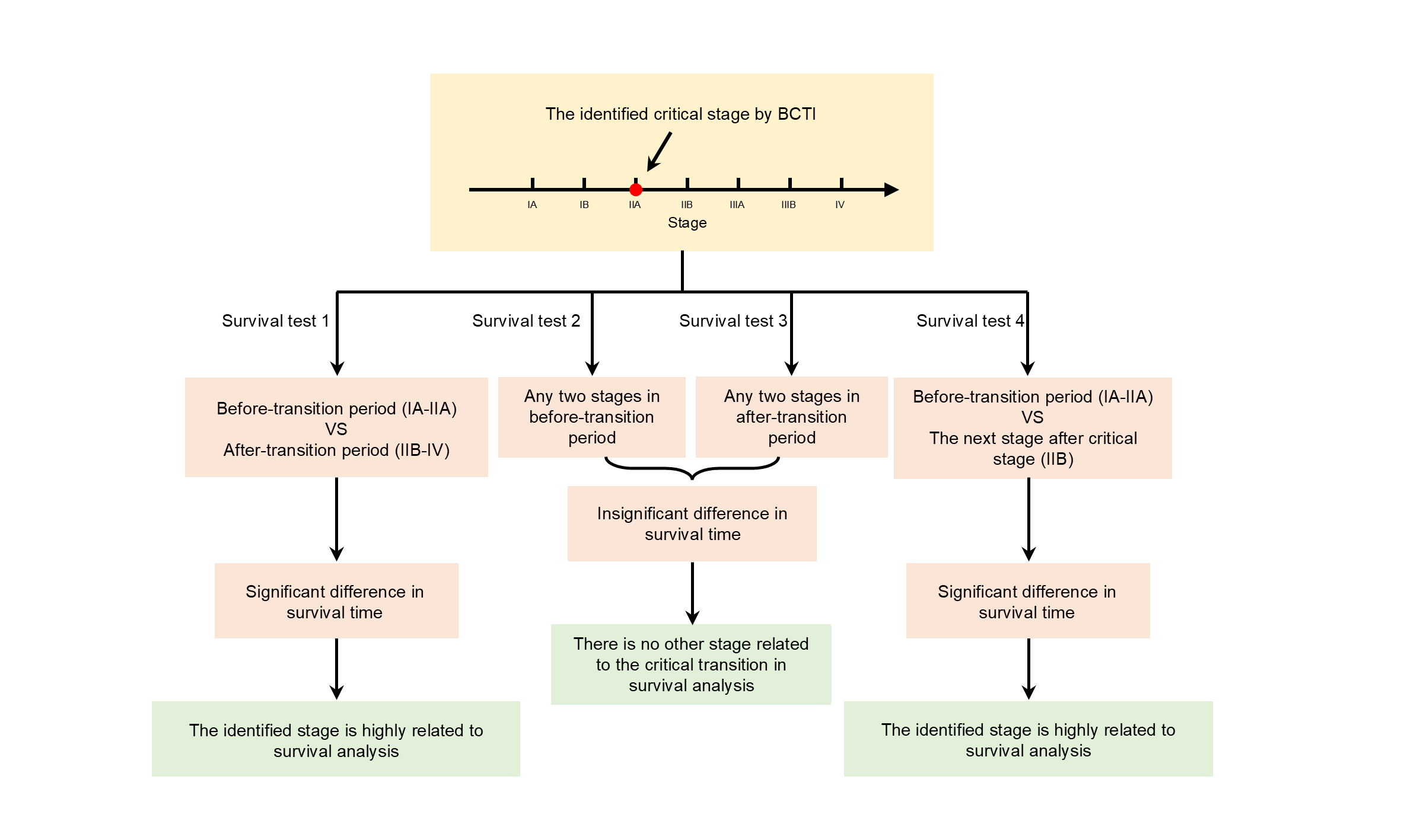


**Figure S11. A schematic illustration for validating the identified critical state.** To validate the identified critical state, the above four steps (survival test 1, survival test 2, survival test 3 and survival test 4) were carried out for validating a critical transition of tumor disease at stage IIA.

## Fig. S12. Validating the identified critical states of THCA


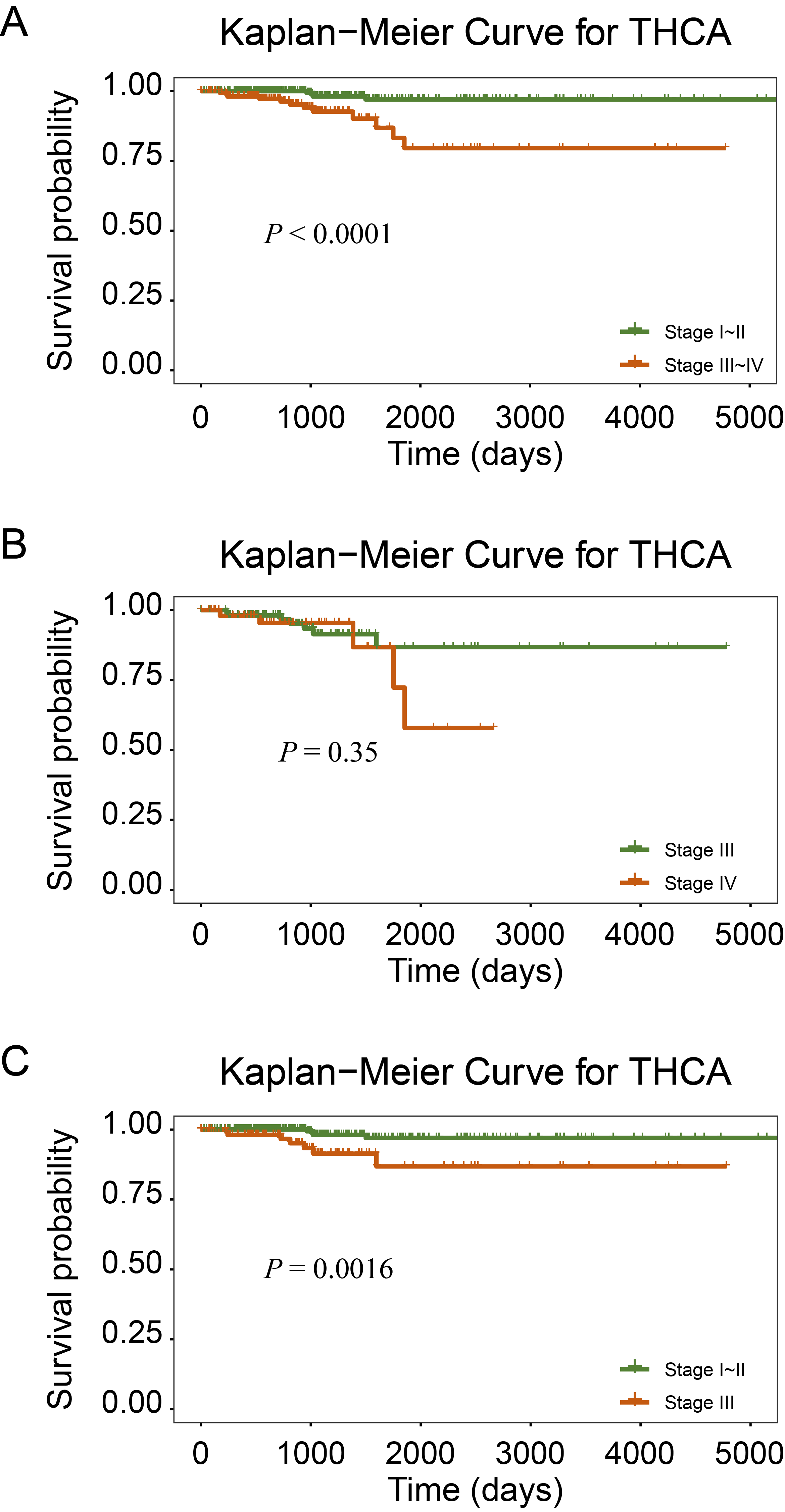


**Figure S12. Validation for the identified critical states of THCA. (A-C)** validate the identified critical state of THCA based on the validation strategy.

## Fig. S13. BCTI uncovered the change of signaling mechanism in the MAPK signaling pathway of THCA


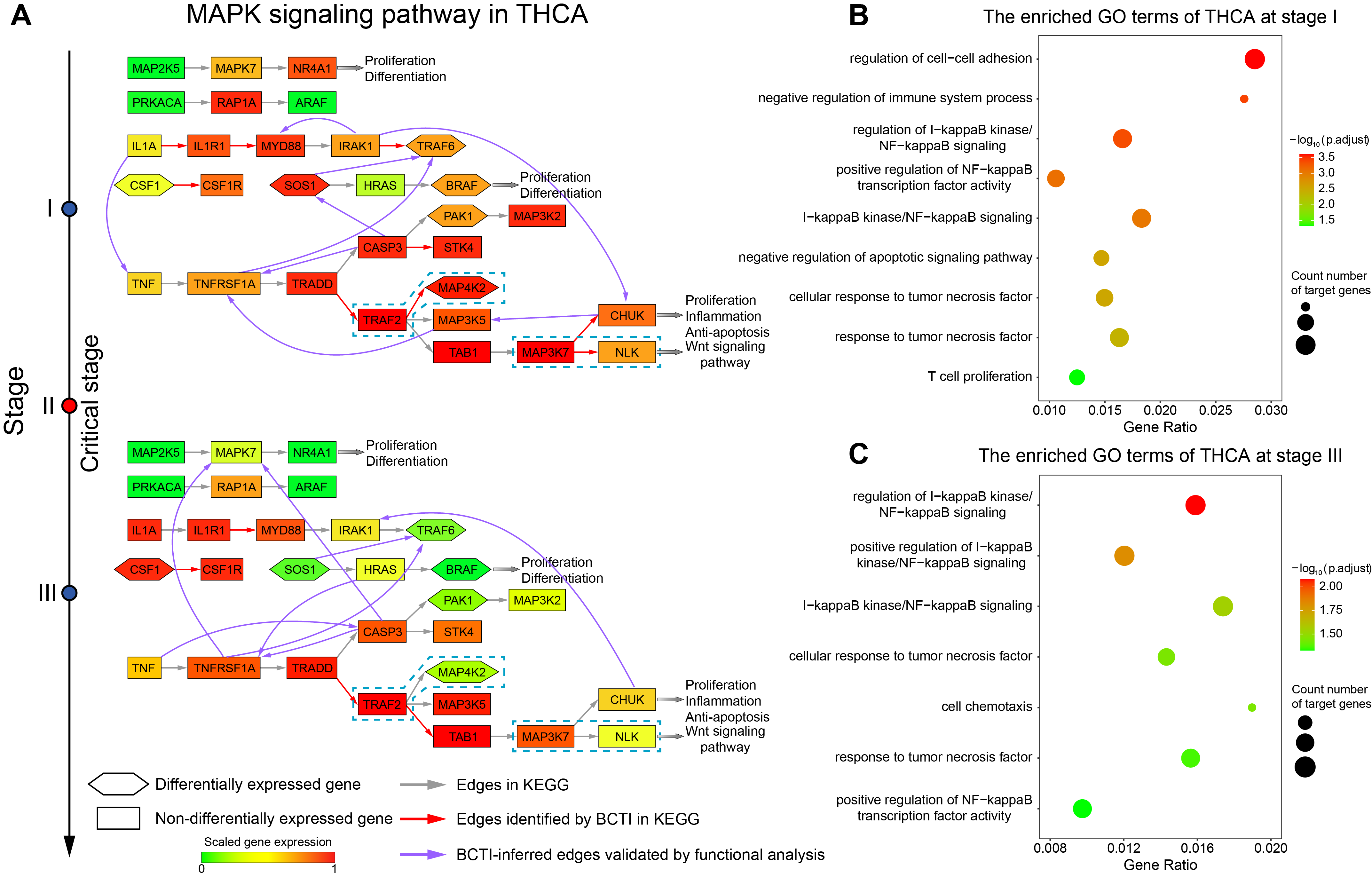


**Figure S13. BCTI uncovered the change of signaling mechanism in the MAPK signaling pathway of THCA. (A)** The change in BCTI’s performance on the gold standard network before and after the critical stage suggests a transition in the underlying signaling mechanism, reflected in the weakening of co-expression patterns between pathway genes after the critical stage. **(B-C)** GO functional enrichment results for the pathway genes at stages I and III, respectively, showing the biological processes significantly associated with the BCTI-inferred regulatory relationships at these stages.

## Fig. S14. Dynamic changes of key hub gene networks before and after the critical transition


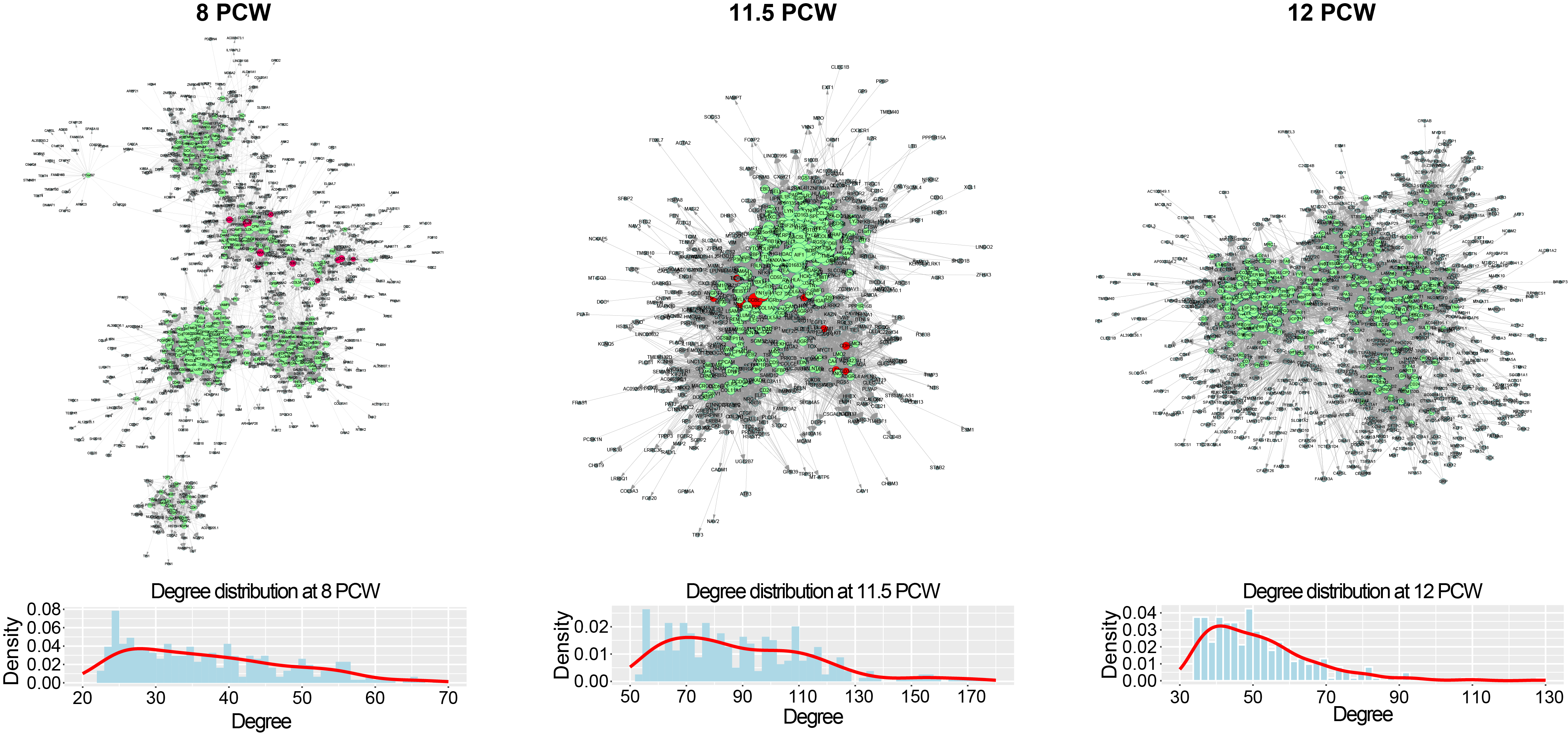


**Figure S14. Dynamic changes of key hub gene networks before and after the critical transition.** Compared to the before-transition-state GRN, the after-transition-state GRNs exhibited tighter connectivity among key hub genes and a higher degree centrality of these genes

**Section S3. The Supplementary Notes**

## Supplementary Note S1. Dynamic network biomarker (DNB) theory

The theoretical foundation for combining the identification of tipping points with gene regulatory network inference is the DNB theory. Specifically, in order to theoretically and mathematically describe the dynamics of a complex disease, its evolution is usually modeled as a time-dependent nonlinear dynamical system [7], where the sudden deterioration is regarded as a state transition at a bifurcation point [8]. Generally, the after-transition state refers to a stable stage after a drastic deterioration. Thus, the after-transition state may be characterized by different deterioration processes (Fig. S2). During the progression of cancer, for instance, it is regarded that the stage of lymphoid infiltrates as the after-transition state after a critical transition of tumor cell invasion. On the other hand, after a critical transition of metastasis, the after-transition state may be taken to represent the stage of being distant metastasis. In an ideal scenario with small noise, when a complex system approaches the critical point, a dominant group of variables, termed DNB biomolecules, becomes apparent among the observed variables. This group meets the following three conditions [9]:

1. The correlation (${PCC}_{\mathrm{in}}$) between any pair of members in the DNB group rapidly increases;
2. The correlation (${PCC}_{\mathrm{out}}$) between one member of the DNB group and any other non-DNB member rapidly decreases;
3. The standard deviation (${SD}_{\mathrm{in}}$) or variation for any member in the DNB group drastically increases.

The above three properties are necessary conditions of the state transition at a codimension-one bifurcation point, and can also be approximately stated as: the occurrence of a group of biomolecules whose expressions are strongly fluctuating and highly correlated, implies an upcoming critical transition. These three properties are the theoretical basis of the DNB method.

To quantify the signal strength of such a transition, a composite indicator known as the Criticality Index (CI) is defined based on the three key properties of the DNB group, as follows:

$$\mathrm{CI} = \frac{(SD \times{PCC}_{in})}{{PCC}_{out}}$$

In this formula, $SD$ refers to the average standard deviation of DNB variables, ${PCC}_{in}$ represents the average Pearson correlation coefficient among members of the DNB group, and ${PCC}_{out}$ denotes the average Pearson correlation coefficient between DNB variables and non-DNB variables. An increasing CI value is typically interpreted as an early-warning signal, suggesting that the system is approaching a critical transition point.

## Supplementary Note S2. Simplifying mutual information calculation with covariance

For a discrete variable $X$, the entropy $\psi(X)$ is the measure of average uncertainty of variable $X$ and can be defined by

$$\begin{aligned} \psi\left( X \right)=-\sum_{x\in X} p\left( x \right)\log p\left( x \right)\text{,}\#\text{(}\text{S}\text{1)} \end{aligned}$$

where $p(x)$ is the probability of each discrete value $x$ in $X$. The joint entropy $\psi(X, Y)$ of $X$ and $Y$ can be denoted by

$$\begin{aligned} \psi\left( X,Y \right)=-\sum_{x\in X,y\in Y} p\left( x,y \right)\log p\left( x,y \right)\text{,}\#\text{(}\text{S}\text{2)} \end{aligned}$$

where $p(x, y)$ is the joint probability of $x$ in $X$ and $y$ in $Y$.

Mutual information (MI) measures the dependency between two variables. For discrete variables $X$ and $Y$, MI is defined as

$$\begin{aligned} I\left( X,Y \right)=-\sum_{x\in X,y\in Y} p\left( x,y \right)\log\frac{p\left( x,y \right)}{p\left( x \right)p\left( y \right)}\text{,}\#\text{(}\text{S}\text{3)} \end{aligned}$$

MI can also be defined in terms of entropies as

$$\begin{aligned} I\left( X,Y \right)=\psi\left( X \right)+\psi\left( Y \right)-\psi\left( X,Y \right)\#\text{(}\text{S}\text{4)} \end{aligned}$$

where $\psi(X, Y)$ is joint entropy of $X$ and $Y$. High MI value indicates that there may be a close relationship between the variables, while low MI value implies their independence. Here, the entropy is estimated with kernel-based fitting methods, such as kernel probability density estimator [10], as follows:

$$\begin{aligned} P\left( X_{i} \right)=\frac{1}{N}\sum_{j=1}^{N} \frac{1}{\left( 2\pi\right)^{\frac{n}{2}}\left| C \right|^{\frac{n}{2}}}\exp\left( -\frac{1}{2}\left( X_{j}-X_{i} \right)^{T}C^{-1}\left( X_{j}-X_{i} \right) \right)\text{,}\#\text{(}\text{S}\text{5)} \end{aligned}$$

where $C$ is the covariance matrix of variable $X$, $\left| C \right|$ is the determinant of matrix $C$, $N$is the number of samples and $n$ is the number of variables in $C$.

With Equations (S1) and (S5), we can get the entropy of variable $X$ as follows.

$$\begin{aligned} \psi\left( X \right)=\log\left[ \left( 2\pi e \right)^{\frac{n}{2}}\left| C \right|^{\frac{1}{2}} \right]=\frac{1}{2}\log\left( 2\pi e \right)^{n}\left| C \right|\text{,}\#\text{(}\text{S}\text{6)} \end{aligned}$$

With Equation (S6), the formulation (S4) can be expressed as follows:

$$\begin{aligned} I\left( X,Y \right)=\frac{1}{2}\log\frac{\left| C\left( X \right) \right|\cdot\left| C\left( Y \right) \right|}{\left| C\left( X,Y \right) \right|}\text{,}\#\text{(}\text{S}\text{7)} \end{aligned}$$

with

$$\left| C\left( X,Y \right) \right|=\left| \begin{matrix} C\left( X \right) & \mathrm{cov}\left( X,Y \right) \\ \mathrm{cov}\left( X,Y \right) & C\left( Y \right) \end{matrix} \right|=\left| C\left( X \right) \right|\cdot\left| C\left( Y \right) \right|\cdot\left| \begin{matrix} 1 & \frac{\mathrm{cov}\left( X,Y \right)}{\left| C\left( X \right) \right|} \\ \frac{\mathrm{cov}\left( X,Y \right)}{\left| C\left( Y \right) \right|} & 1 \end{matrix} \right|$$

$$\begin{aligned} =\left| C\left( X \right) \right|\cdot\left| C\left( Y \right) \right|\cdot\left( 1-\left( \frac{\mathrm{cov}\left( X,Y \right)}{\sqrt{\left| C\left( X \right) \right|\cdot\left| C\left( Y \right) \right|}} \right)^{2} \right)=\left| C\left( X \right) \right|\cdot\left| C\left( Y \right) \right|\left( 1-\rho^{2} \right)\text{,}\#\text{(S8)} \end{aligned}$$

where $\rho$ represents the Pearson correlation coefficient of $X$ and $Y$, and $\mathrm{cov}\left( X,Y \right)$ is the covariance between the variables $X$ and $Y$. Thus, the formulation (S4) can be expressed as $I\left( X,Y \right)=-\frac{1}{2}\log\left( 1-\rho^{2} \right)$. To prevent numerical instability when $\rho$ approaches 1, a small constant ($\varepsilon={10}^{-5}$) was added inside the logarithmic term during the computation. Therefore, the final formulation during the computation is expressed as $I\left( X,Y \right)=-\frac{1}{2}\log\left( 1-\rho^{2}+\varepsilon\right)$.

## Supplementary Note S3. Explanation for the relationship between the $\boldsymbol{H}$ score and tipping point

To theoretically explain how the $H$ score works in detecting the tipping point, we denote the original multiple linear regression problem in the following form:

$$\begin{aligned} \boldsymbol{y}=\boldsymbol{\beta}X+\boldsymbol{e}\text{,}\#\text{(}\text{S9}\text{)} \end{aligned}$$

where $\boldsymbol{y\in}\mathbb{R}^{1\times n}$ is a vector containing $n$ observations of a random variable $Y$, $X\in\mathbb{R}^{p\times n}$ is a matrix containing $p\times n$ observations of $p$ regressors (variables) (*i.e.*, $X_{1},X_{2},\ldots,X_{p}$), $\boldsymbol{\beta\in}\mathbb{R}^{1\times p}$ is a vector with $p$ population parameters, and $\boldsymbol{e\in}\mathbb{R}^{1\times n}$ is a $n$-dimensional vector containing $n$ error values of the error variable $E$ with $E\boldsymbol{\sim}N\left( 0,\sigma^{2} \right)$. Since the input data have been standardized using z-score normalization, all variables now have a mean of 0 and a standard deviation of 1. In this study, assuming that $rank\left( X \right)=p$, we can employ the least squares method to solve the system of linear equations, *i.e.*, $\boldsymbol{y}=\boldsymbol{\beta}X$, thereby obtaining the unique estimate for $\boldsymbol{\beta}$, denoted as $\hat{\boldsymbol{\beta}}\boldsymbol{=}\left( {\boldsymbol{y}X}^{'} \right)\left( {XX}^{'} \right)^{-1}$, where the symbol “ $'$ ” represents the transpose for a matrix or vector, and “ ${(\cdot)}^{-1}$ ” represents the inverse for a matrix. Specifically,

$$X=\left( \begin{matrix} x_{11} & x_{12} & \cdots& x_{1n} \\ x_{21} & x_{22} & \cdots& x_{2n} \\ \vdots& \vdots& \ddots& \vdots\\ x_{p1} & x_{p2} & \cdots& x_{pn} \end{matrix} \right)_{p\times n}$$

$$\boldsymbol{y}=\left( y_{1},\cdots,y_{n} \right)_{1\times n}$$

$$\hat{\boldsymbol{\beta}}=\left( \boldsymbol{y}X_{n\times p}^{\boldsymbol{'}} \right)\left( X_{p\times n}X_{n\times p}^{\boldsymbol{'}} \right)^{-1}$$

$\begin{aligned} =\left[ \left( y_{1},\cdots,y_{n} \right)\cdot\left( \begin{matrix} x_{11} & x_{21} & \cdots& x_{p1} \\ x_{12} & x_{22} & \cdots& x_{p2} \\ \vdots& \vdots& \ddots& \vdots\\ x_{1n} & x_{2n} & \cdots& x_{pn} \end{matrix} \right) \right]\cdot\\ \left[ \left( \begin{matrix} x_{11} & x_{12} & \cdots& x_{1n} \\ x_{21} & x_{22} & \cdots& x_{2n} \\ \vdots& \vdots& \ddots& \vdots\\ x_{p1} & x_{p2} & \cdots& x_{pn} \end{matrix} \right)\cdot\left( \begin{matrix} x_{11} & x_{21} & \cdots& x_{p1} \\ x_{12} & x_{22} & \cdots& x_{p2} \\ \vdots& \vdots& \ddots& \vdots\\ x_{1n} & x_{2n} & \cdots& x_{pn} \end{matrix} \right) \right]^{-1}\text{.}\#\text{(}\text{S10}\text{)} \end{aligned}$

According to the definition of covariance and the property of z-score normalization, the mathematical formulation of $\hat{\boldsymbol{\beta}}$ can be transformed as follows:

$$\begin{aligned} \hat{\boldsymbol{\beta}}=\boldsymbol{C}_{YX}\cdot C_{XX}^{-1}\text{,}\#\text{(}\text{S11}\text{)} \end{aligned}$$

where:

${\boldsymbol{C}_{YX}=\left[ \mathrm{cov}(Y,X_{1}),\cdots,\mathrm{cov}(Y,X_{p}) \right]}_{1\times p}$,

$C_{XX}=\left[ \begin{matrix} \mathrm{cov}(X_{1},X_{1}) & \mathrm{cov}(X_{1},X_{2}) & \cdots& \mathrm{cov}(X_{1},X_{p}) \\ \mathrm{cov}(X_{2},X_{1}) & \mathrm{cov}(X_{2},X_{2}) & \cdots& \mathrm{cov}(X_{2},X_{p}) \\ \vdots& \vdots& \ddots& \vdots\\ \mathrm{cov}(X_{p},X_{1}) & \mathrm{cov}(X_{p},X_{2}) & \cdots& \mathrm{cov}(X_{p},X_{p}) \end{matrix} \right]_{p\times p}$,

$\mathrm{cov}(\cdot,\cdot)$ represents the covariance between two random variables. Thus, the transpose of $\hat{\boldsymbol{\beta}}$ is denoted as ${\hat{\boldsymbol{\beta}}}^{\boldsymbol{'}}=C_{XX}^{-1}\cdot\boldsymbol{C}_{YX}^{'}$. Meanwhile, we can obtain the predicted vector of the variable $Y$ based on $\hat{\boldsymbol{\beta}}$, denoted as $\hat{\boldsymbol{y}}=\hat{\boldsymbol{\beta}}X$. Subsequently, the error between the predicted and true values is calculated as follows:

$$\mathrm{RMSE}(\boldsymbol{y,}\hat{\boldsymbol{y}})=\left\| \boldsymbol{y}-\hat{\boldsymbol{y}} \right\|_{2}=\left( \boldsymbol{y}-\hat{\boldsymbol{y}} \right)\cdot\left( \boldsymbol{y}-\hat{\boldsymbol{y}} \right)^{'}=\boldsymbol{y}\cdot\boldsymbol{y}^{'}-\boldsymbol{y}\cdot{\hat{\boldsymbol{y}}}^{'}-\hat{\boldsymbol{y}}\cdot\boldsymbol{y}^{'}+\hat{\boldsymbol{y}}\cdot{\hat{\boldsymbol{y}}}^{'}$$

$$=\left( y_{1},\cdots,y_{n} \right)_{1\times n}\cdot\left( \begin{matrix} y_{1} \\ \vdots\\ y_{n} \end{matrix} \right)_{n\times1}-\left( y_{1},\cdots,y_{n} \right)_{1\times n}\cdot X^{'}{\hat{\boldsymbol{\beta}}}^{'}-\hat{\boldsymbol{\beta}}X\cdot\left( \begin{matrix} y_{1} \\ \vdots\\ y_{n} \end{matrix} \right)_{n\times1}+\hat{\boldsymbol{\beta}}X\cdot X^{'}{\hat{\boldsymbol{\beta}}}^{'}$$

$$=n\cdot\mathrm{Var}\left( Y \right)-\left( y_{1},\cdots,y_{n} \right)_{1\times n}\cdot\left( \begin{matrix} x_{11} & x_{21} & \cdots& x_{p1} \\ x_{12} & x_{22} & \cdots& x_{p2} \\ \vdots& \vdots& \ddots& \vdots\\ x_{1n} & x_{2n} & \cdots& x_{pn} \end{matrix} \right)_{n\times p}\cdot{\hat{\boldsymbol{\beta}}}^{'}-\hat{\boldsymbol{\beta}}\cdot\left( \begin{matrix} x_{11} & x_{12} & \cdots& x_{1n} \\ x_{21} & x_{22} & \cdots& x_{2n} \\ \vdots& \vdots& \ddots& \vdots\\ x_{p1} & x_{p2} & \cdots& x_{pn} \end{matrix} \right)_{p\times n}\cdot\left( \begin{matrix} y_{1} \\ \vdots\\ y_{n} \end{matrix} \right)_{n\times1}+\hat{\boldsymbol{\beta}}\cdot\left( \begin{matrix} x_{11} & x_{12} & \cdots& x_{1n} \\ x_{21} & x_{22} & \cdots& x_{2n} \\ \vdots& \vdots& \ddots& \vdots\\ x_{p1} & x_{p2} & \cdots& x_{pn} \end{matrix} \right)_{p\times n}\cdot\left( \begin{matrix} x_{11} & x_{21} & \cdots& x_{p1} \\ x_{12} & x_{22} & \cdots& x_{p2} \\ \vdots& \vdots& \ddots& \vdots\\ x_{1n} & x_{2n} & \cdots& x_{pn} \end{matrix} \right)_{n\times p}\cdot{\hat{\boldsymbol{\beta}}}^{'}$$

$$=n\cdot\mathrm{Var}\left( Y \right)-\left[ n\mathrm{cov}\left( Y,X_{1} \right),\cdots,n\mathrm{cov}\left( Y,X_{p} \right) \right]_{1\times p}\cdot{\hat{\boldsymbol{\beta}}}^{'}-\hat{\boldsymbol{\beta}}\cdot\left[ \begin{matrix} n\mathrm{cov}\left( X_{1},Y \right) \\ \vdots\\ n\mathrm{cov}\left( X_{p},Y \right) \end{matrix} \right]_{p\times1}$$

$$+\hat{\boldsymbol{\beta}}\cdot\left[ \begin{matrix} \mathrm{cov}(X_{1},X_{1}) & \mathrm{cov}(X_{1},X_{2}) & \cdots& \mathrm{cov}(X_{1},X_{p}) \\ \mathrm{cov}(X_{2},X_{1}) & \mathrm{cov}(X_{2},X_{2}) & \cdots& \mathrm{cov}(X_{2},X_{p}) \\ \vdots& \vdots& \ddots& \vdots\\ \mathrm{cov}(X_{p},X_{1}) & \mathrm{cov}(X_{p},X_{2}) & \cdots& \mathrm{cov}(X_{p},X_{p}) \end{matrix} \right]_{p\times p}\cdot{\hat{\boldsymbol{\beta}}}^{'}$$

$$=n\cdot\mathrm{Var}\left( Y \right)-n\boldsymbol{C}_{YX}\cdot\left( C_{XX}^{-1}\cdot\boldsymbol{C}_{YX}^{'} \right)-\left( \boldsymbol{C}_{YX}\cdot C_{XX}^{-1} \right)\cdot n\boldsymbol{C}_{YX}^{'}$$

$$+\left( \boldsymbol{C}_{YX}\cdot C_{XX}^{-1} \right)\cdot nC_{XX}\cdot\left( C_{XX}^{-1}\cdot\boldsymbol{C}_{YX}^{'} \right)$$

$$\begin{aligned} =n\cdot\mathrm{Var}\left( Y \right)-n\boldsymbol{C}_{YX}\cdot C_{XX}^{-1}\cdot\boldsymbol{C}_{YX}^{'}\text{,}\#\text{(}\text{S12}\text{)} \end{aligned}$$

where $\mathrm{Var}\left( Y \right)$ represents the variance of the variable $Y$. According to the definition of Pearson correlation coefficient and its relationship to covariance, we can transform the above covariance matrices/vectors as follows:

$$C_{XX}=\left[ \begin{matrix} \mathrm{cov}(X_{1},X_{1}) & \mathrm{cov}(X_{1},X_{2}) & \cdots& \mathrm{cov}(X_{1},X_{p}) \\ \mathrm{cov}(X_{2},X_{1}) & \mathrm{cov}(X_{2},X_{2}) & \cdots& \mathrm{cov}(X_{2},X_{p}) \\ \vdots& \vdots& \ddots& \vdots\\ \mathrm{cov}(X_{p},X_{1}) & \mathrm{cov}(X_{p},X_{2}) & \cdots& \mathrm{cov}(X_{p},X_{p}) \end{matrix} \right]_{p\times p}$$

$$=\left[ \begin{matrix} \mathrm{SD}\left( X_{1} \right)\mathrm{SD}\left( X_{1} \right)\mathrm{PCC}(X_{1},X_{1}) & \mathrm{SD}\left( X_{1} \right)\mathrm{SD}\left( X_{2} \right)\mathrm{PCC}(X_{1},X_{2}) & \cdots& \mathrm{SD}\left( X_{1} \right)\mathrm{SD}\left( X_{p} \right)\mathrm{PCC}(X_{1},X_{p}) \\ \mathrm{SD}\left( X_{2} \right)\mathrm{SD}\left( X_{1} \right)\mathrm{PCC}(X_{2},X_{1}) & \mathrm{SD}\left( X_{2} \right)\mathrm{SD}\left( X_{2} \right)\mathrm{PCC}(X_{2},X_{2}) & \cdots& \mathrm{SD}\left( X_{2} \right)\mathrm{SD}\left( X_{p} \right)\mathrm{PCC}(X_{2},X_{p}) \\ \vdots& \vdots& \ddots& \vdots\\ \mathrm{SD}\left( X_{p} \right)\mathrm{SD}\left( X_{1} \right)\mathrm{PCC}(X_{p},X_{1}) & \mathrm{SD}\left( X_{p} \right)\mathrm{SD}\left( X_{2} \right)\mathrm{PCC}(X_{p},X_{2}) & \cdots& \mathrm{SD}\left( X_{p} \right)\mathrm{SD}\left( X_{p} \right)\mathrm{PCC}(X_{p},X_{p}) \end{matrix} \right]_{p\times p}$$

$$=\left[ \begin{matrix} \mathrm{SD}\left( X_{1} \right) & 0 & \cdots& 0 \\ 0 & \mathrm{SD}\left( X_{2} \right) & \cdots& 0 \\ \vdots& \vdots& \ddots& \vdots\\ 0 & 0 & \cdots& \mathrm{SD}\left( X_{p} \right) \end{matrix} \right]_{p\times p}\cdot\left[ \begin{matrix} \mathrm{PCC}\left( X_{1},X_{1} \right) & \mathrm{PCC}\left( X_{1},X_{2} \right) & \cdots& \mathrm{PCC}\left( X_{1},X_{p} \right) \\ \mathrm{PCC}\left( X_{2},X_{1} \right) & \mathrm{PCC}\left( X_{2},X_{2} \right) & \cdots& \mathrm{PCC}\left( X_{2},X_{p} \right) \\ \vdots& \vdots& \ddots& \vdots\\ \mathrm{PCC}\left( X_{p},X_{1} \right) & \mathrm{PCC}\left( X_{p},X_{2} \right) & \cdots& \mathrm{PCC}\left( X_{p},X_{p} \right) \end{matrix} \right]_{p\times p}\cdot$$

$$\begin{aligned} \left[ \begin{matrix} \mathrm{SD}\left( X_{1} \right) & 0 & \cdots& 0 \\ 0 & \mathrm{SD}\left( X_{2} \right) & \cdots& 0 \\ \vdots& \vdots& \ddots& \vdots\\ 0 & 0 & \cdots& \mathrm{SD}\left( X_{p} \right) \end{matrix} \right]_{p\times p}\text{,}\#\text{(}\text{S13}\text{)} \end{aligned}$$

$\boldsymbol{C}_{YX}=\left[ \mathrm{cov}(Y,X_{1}),\cdots,\mathrm{cov}(Y,X_{p}) \right]_{1\times p}$

$$=\left[ \mathrm{SD}\left( Y \right)\mathrm{SD}\left( X_{1} \right)\mathrm{PCC}(Y,X_{1}),\cdots,\mathrm{SD}\left( Y \right)\mathrm{SD}\left( X_{p} \right)\mathrm{PCC}(Y,X_{p}) \right]_{1\times p}$$

$$\begin{aligned} =\mathrm{SD}\left( Y \right)\left[ \mathrm{PCC}(Y,X_{1}),\cdots,\mathrm{PCC}(Y,X_{p}) \right]_{1\times p}\left[ \begin{matrix} \mathrm{SD}\left( X_{1} \right) & 0 & \cdots& 0 \\ 0 & \mathrm{SD}\left( X_{2} \right) & \cdots& 0 \\ \vdots& \vdots& \ddots& \vdots\\ 0 & 0 & \cdots& \mathrm{SD}\left( X_{p} \right) \end{matrix} \right]_{p\times p}\text{,}\#\text{(}\text{S14}\text{)} \end{aligned}$$

where $\mathrm{PCC}(\cdot,\cdot)$ represents the Pearson correlation coefficient between two random variables, and $\mathrm{SD}(\cdot)$ represents the standard deviation. Therefore, $\mathrm{RMSE}(\boldsymbol{y,}\hat{\boldsymbol{y}})$ can be transformed as follows:

$$\mathrm{RMSE}\left( \boldsymbol{y,}\hat{\boldsymbol{y}} \right)=n\cdot\mathrm{Var}\left( \boldsymbol{y} \right)-n\boldsymbol{C}_{YX}\cdot C_{XX}^{-1}\cdot\boldsymbol{C}_{YX}^{'}$$

$$=n\cdot\mathrm{Var}\left( Y \right)-n\cdot\mathrm{SD}\left( Y \right)\left[ \mathrm{PCC}(Y,X_{1}),\cdots,\mathrm{PCC}(Y,X_{p}) \right]_{1\times p}\left[ \begin{matrix} \mathrm{SD}\left( X_{1} \right) & 0 & \cdots& 0 \\ 0 & \mathrm{SD}\left( X_{2} \right) & \cdots& 0 \\ \vdots& \vdots& \ddots& \vdots\\ 0 & 0 & \cdots& \mathrm{SD}\left( X_{p} \right) \end{matrix} \right]_{p\times p}$$

$$\cdot\left[ \begin{matrix} \mathrm{SD}\left( X_{1} \right) & 0 & \cdots& 0 \\ 0 & \mathrm{SD}\left( X_{2} \right) & \cdots& 0 \\ \vdots& \vdots& \ddots& \vdots\\ 0 & 0 & \cdots& \mathrm{SD}\left( X_{p} \right) \end{matrix} \right]_{p\times p}^{-1}\cdot\left[ \begin{matrix} \mathrm{PCC}(X_{1},X_{1}) & \mathrm{PCC}(X_{1},X_{2}) & \cdots& \mathrm{PCC}(X_{1},X_{p}) \\ \mathrm{PCC}(X_{2},X_{1}) & \mathrm{PCC}(X_{2},X_{2}) & \cdots& \mathrm{PCC}(X_{2},X_{p}) \\ \vdots& \vdots& \ddots& \vdots\\ \mathrm{PCC}(X_{p},X_{1}) & \mathrm{PCC}(X_{p},X_{2}) & \cdots& \mathrm{PCC}(X_{p},X_{p}) \end{matrix} \right]_{p\times p}^{-1}$$

$$\cdot\left[ \begin{matrix} \mathrm{SD}\left( X_{1} \right) & 0 & \cdots& 0 \\ 0 & \mathrm{SD}\left( X_{2} \right) & \cdots& 0 \\ \vdots& \vdots& \ddots& \vdots\\ 0 & 0 & \cdots& \mathrm{SD}\left( X_{p} \right) \end{matrix} \right]_{p\times p}^{-1}\cdot\mathrm{SD}\left( Y \right)\left[ \begin{matrix} \mathrm{SD}\left( X_{1} \right) & 0 & \cdots& 0 \\ 0 & \mathrm{SD}\left( X_{2} \right) & \cdots& 0 \\ \vdots& \vdots& \ddots& \vdots\\ 0 & 0 & \cdots& \mathrm{SD}\left( X_{p} \right) \end{matrix} \right]_{p\times p}$$

$$\cdot\left[ \begin{aligned} \mathrm{PCC}\left( X_{1},Y \right) \\ \vdots\\ \mathrm{PCC}\left( X_{p},Y \right) \end{aligned} \right]_{p\times1}= n\cdot\mathrm{Var}\left( Y \right)-n\cdot\mathrm{Var}\left( Y \right)\left[ \mathrm{PCC}(Y,X_{1}),\cdots,\mathrm{PCC}(Y,X_{p}) \right]_{1\times p}$$

$\cdot\left[ \begin{matrix} \mathrm{PCC}(X_{1},X_{1}) & \mathrm{PCC}(X_{1},X_{2}) & \cdots& \mathrm{PCC}(X_{1},X_{p}) \\ \mathrm{PCC}(X_{2},X_{1}) & \mathrm{PCC}(X_{2},X_{2}) & \cdots& \mathrm{PCC}(X_{2},X_{p}) \\ \vdots& \vdots& \ddots& \vdots\\ \mathrm{PCC}(X_{p},X_{1}) & \mathrm{PCC}(X_{p},X_{2}) & \cdots& \mathrm{PCC}(X_{p},X_{p}) \end{matrix} \right]_{p\times p}^{-1}\cdot\left[ \begin{aligned} \mathrm{PCC}\left( X_{1},Y \right) \\ \vdots\\ \mathrm{PCC}\left( X_{p},Y \right) \end{aligned} \right]_{p\times1}$

$$\begin{aligned} =n\cdot\mathrm{Var}\left( Y \right)\cdot W\text{,}\#\text{(}\text{S15}\text{)} \end{aligned}$$

where:

$$\mathbf{PCC}_{YX}=\left[ \mathrm{PCC}(Y,X_{1}),\cdots,\mathrm{PCC}(Y,X_{p}) \right]_{1\times p}$$

$${PCC}_{XX}=\left[ \begin{matrix} \mathrm{PCC}(X_{1},X_{1}) & \mathrm{PCC}(X_{1},X_{2}) & \cdots& \mathrm{PCC}(X_{1},X_{p}) \\ \mathrm{PCC}(X_{2},X_{1}) & \mathrm{PCC}(X_{2},X_{2}) & \cdots& \mathrm{PCC}(X_{2},X_{p}) \\ \vdots& \vdots& \ddots& \vdots\\ \mathrm{PCC}(X_{p},X_{1}) & \mathrm{PCC}(X_{p},X_{2}) & \cdots& \mathrm{PCC}(X_{p},X_{p}) \end{matrix} \right]_{p\times p}$$

$$\mathbf{PCC}_{XY}=\left[ \begin{aligned} \mathrm{PCC}\left( X_{1},Y \right) \\ \vdots\\ \mathrm{PCC}\left( X_{p},Y \right) \end{aligned} \right]_{p\times1}$$

$$W=\left( 1-\mathbf{PCC}_{YX}\cdot{PCC}_{XX}^{-1}\cdot\mathbf{PCC}_{XY} \right)$$

According to the DNB theory described in Supplementary Note S1, when the system approaches a critical point, a group of DNBs emerges, exhibiting a sharp increase in PCC among them and fluctuations in gene expression, while the PCC between DNBs and non-DNBs sharply decreases. According to the conclusion derived in Supplementary Note S2, a sharp increase in PCC indicates a marked rise in mutual information. When constructing the correlation basis network (CBN), we filter out the adjacent nodes with low mutual information for each gene and retain only interactions between genes with high mutual information, thereby meeting the first condition of the DNB theory. As a result, Therefore, on one hand, gene expression fluctuations sharply increase at the critical point, as explained by the DNB theory. On the other hand, there is an inherent positive correlation between gene expression fluctuations and the $H$ score (Eq. (S15)). Together, these two factors contribute to a rapid rise in the residual sum of squares, serving as an early warning of an impending critical state.

## Supplementary Note S4. One-sample *t*-test

The one-sample *t*-test [11] is employed to determine whether constant $x$ is statistically significantly different from the mean of an $n$-dimensional vector $\boldsymbol{X}=(x_{1},x_{2},\cdots,x_{n})$. The one-sample *t*-test statistic is defined by the following equation:

$$\begin{aligned} ST=\frac{\bar{X}-x}{SD(\boldsymbol{X})/\sqrt{n}}\text{,}\#\text{(}\text{S16}\text{)} \end{aligned}$$

where $\bar{X}$ represents the mean of vector $\boldsymbol{X}$ and $SD(\boldsymbol{X})$ represents the standard deviation of vector $\boldsymbol{X}$. The statistical index $S$quantitatively measures the significant difference between $\bar{X}$ and $x$. To estimate the statistical significance, the P-value $P$ (the probability associated with $ST$) can be obtained by the $t$-distribution. There is a significant difference between $\bar{X}$and $x$ if $P<0.05$, otherwise, the difference is not significant. In this study, to accurately analyze the dynamic process of complex diseases based on the BCTI score, we utilize the above strategy to confirm the appearance of the critical point. The time point $T=t$ is considered to be a critical point if there is a significant difference between the current BCTI score $S(t)$ and the mean value of a vector $(S\left( 1 \right),S\left( 2 \right),\ldots,S\left( t-1 \right))$ ($P<0.05$). In particular, when $t=2$, the time point $T=t$ is considered a critical point if $S\left( t \right)$ is significantly different from the mean of vector $(S(1), S(3))$.

## Supplementary Note S5. Details for the data and benchmark methods description

Specifically, the DREAM challenge dataset is widely used for gene regulation prediction. It includes gene expression data generated by computer simulation, which typically encompasses datasets of various sizes and standard networks that have been validated in *E. coli* and yeast. The IRMA dataset is a synthetic non-linear time series network embedded in the *Saccharomyces cerevisiae* genome, comprising 5 genes and 6 regulation edges. The simulated sixteen-node network represents a regulatory network with a system of stochastic differential equations in the Michaelis‒Menten form, wherein the bifurcation $q = 0$ is employed to model the state transition of the network. The SOS DNA repair experimental data is real *E. coli* gene expression data, and its network structure has been verified by real experiments, consisting of 9 genes and 24 regulatory interactions. Moreover, BCTI was applied to the TCGA datasets, downloaded from the University of California Santa Cruz (UCSC) Xena platform (https://xenabrowser.net), to predict the regulatory relationships and detect tipping points within three cancer expression datasets: COAD, LUAD, and THCA datasets. More details for the datasets are provided in Tables S1 and S2.

To benchmark the performance of BCTI on the GRN inference, we compared it with several well-known methods on the aforementioned datasets, including GENIE3, GENIMS, GNIPLR, KBoost, NARROMI, NIMEFI and PLSET. GENIE3 is a GRN inference method based on variable selection with ensembles of regression trees [12]. GENIMS decomposes the gene network inference problem into individual regression problems and solved them with the guided regularized random forest algorithm [13]. GNIPLR adopts projection and lagged regression strategies to infer GRNs [14]. KBoost employs kernel PCA regression, boosting and Bayesian model averaging for reconstruction of GRNs [15]. NARROMI combines ordinary differential equation-based recursive optimization and information theory-based mutual information to improve the accuracy of GRN inference [16]. NIMEFI is a subsampling approach which allows any feature selection algorithm that can produce a ranking to be cast into an ensemble feature importance scoring algorithm [17]. PLSET expresses the GRN inference problem as a feature selection problem and solves it with the partial least squares-based feature selection method combined with a statistical technique for refining the predictions [18]. For most of the methods in comparison, we used the default parameters to run them. For instance, we set the width parameter of the RBF Kernel in KBoost to 40.

Furthermore, the GRN inference performance was evaluated by several statistical indicators, including true positive (TP), false positive (FP), false negative (FN), true negative (TN), precision, true positive rate (TPR), false positive rate (FPR), specificity and accuracy metrics. The mathematical definition can be represented as follows:

$$\begin{aligned} \mathrm{Precision}= \frac{\mathrm{TP}}{TP+FP}\text{,}\#\text{(}\text{S17}\text{)} \end{aligned}$$

$$\begin{aligned} \mathrm{TPR}= \frac{\mathrm{TP}}{TP+FN}\text{,}\#\text{(}\text{S18}\text{)} \end{aligned}$$

$$\begin{aligned} \mathrm{FPR}= \frac{\mathrm{FP}}{FP+TN}\text{,}\#\text{(}\text{S19}\text{)} \end{aligned}$$

$$\begin{aligned} \mathrm{Specificity}= \frac{\mathrm{TN}}{FP+TN}\text{,}\#\text{(}\text{S20}\text{)} \end{aligned}$$

$$\begin{aligned} \mathrm{ACC}= \frac{TP+TN}{P+N}\text{,}\#\text{(}\text{S21}\text{)} \end{aligned}$$

where precision is the proportion of true positive predictions in all positive predictions. TPR refers to the rate of true positive predictions out of all positive cases. Correspondingly, FPR is calculated as the ratio between the number of negative events wrongly categorized as positive (false positives) and the total number of actual negative events. Specificity means the rate of true negative predictions among all negative cases. Accuracy represents the rate of true positive predictions and true negative predictions out of all positive cases and all negative cases.

## Supplementary Note S6. Details of numerical simulation

We use a sixteen-gene network to conduct a numerical simulation and theoretically demonstrate the detection of early-warning signals through the BCTI algorithm [19]. The following sixteen differential equations represent the gene regulation of sixteen genes in a network where gene regulation is represented in a Michaelis-Menten form with the change rates, which are often used to study various biological processes including transcription, translation, diffusion, and translocation processes [20-24]. The differential equation set is as follows:

$$\begin{aligned} \left\{ \begin{aligned} &\frac{dz_{1}(t)}{dt}=\frac{(8-4q)z_{2}(t)}{15(1+z_{2}(t))}-\frac{4\left( 1+q \right)}{15}z_{1}\left( t \right)+\zeta_{1}(t) \\ &\frac{dz_{2}(t)}{dt}=\frac{(4-2q)z_{1}(t)}{15(1+z_{1}(t))}-\frac{2\left( 1+q \right)}{15}z_{2}\left( t \right)+\zeta_{2}(t) \\ &\frac{dz_{3}(t)}{dt}=\frac{4q-10}{15}+\frac{5-2q}{15(1+z_{1}(t))}+\frac{5-2q}{15(1+z_{2}(t))}-z_{3}\left( t \right)+\zeta_{3}(t) \\ &\frac{dz_{4}(t)}{dt}=\frac{(6-2q)z_{1}(t)}{15(1+z_{1}(t))}+\frac{(6-2q)z_{2}(t)}{15(1+z_{2}(t))}-\frac{6}{5}z_{4}\left( t \right)+\zeta_{4}(t) \\ &\frac{dz_{5}(t)}{dt}=\frac{4q-14}{15}+\frac{7-2q}{15(1+z_{1}(t))}+\frac{7-2q}{15(1+z_{2}(t))}-\frac{7}{5}z_{5}\left( t \right)+\zeta_{5}(t) \\ &\frac{dz_{6}(t)}{dt}=\frac{4q-16}{15}+\frac{2(4-2q)}{15(1+z_{1}(t))}+\frac{2(4-2q)}{15(1+z_{2}(t))}-\frac{8}{5}z_{6}\left( t \right)+\zeta_{6}(t) \\ &\frac{dz_{7}(t)}{dt}=\frac{(9-2q)z_{1}(t)}{15(1+z_{1}(t))}+\frac{(9-2q)z_{2}(t)}{15(1+z_{2}(t))}-\frac{9}{5}z_{7}\left( t \right)+\zeta_{7}(t) \\ &\frac{dz_{8}\left( t \right)}{dt}=-\frac{13}{15}+\frac{2}{15\left( 1+z_{1}\left( t \right) \right)}+\frac{2}{15\left( 1+z_{2}\left( t \right) \right)}+\frac{2}{5\left( 1+z_{6}\left( t \right) \right)}+\frac{2z_{10}\left( t \right)}{5\left( 1+z_{10}\left( t \right) \right)} \\ +\frac{3z_{12}\left( t \right)}{5\left( 1+z_{12}\left( t \right) \right)}+\frac{z_{15}\left( t \right)}{5\left( 1+z_{15}\left( t \right) \right)}+\frac{1}{5\left( 1+z_{16}\left( t \right) \right)}-2z_{8}\left( t \right)+\zeta_{8}\left( t \right) \\ &\frac{dz_{9}\left( t \right)}{dt}=-1+\frac{1}{5\left( 1+z_{1}\left( t \right) \right)}+\frac{1}{5\left( 1+z_{2}\left( t \right) \right)}+\frac{3}{5\left( 1+z_{6}\left( t \right) \right)}-\frac{11}{5}z_{9}\left( t \right)+\zeta_{9}(t) \\ &\frac{dz_{10}\left( t \right)}{dt}=\frac{3z_{12}\left( t \right)}{5\left( 1+z_{12}\left( t \right) \right)}-\frac{12}{5}z_{10}\left( t \right)+\zeta_{10}(t) \\ &\frac{dz_{11}\left( t \right)}{dt}=\frac{z_{12}\left( t \right)}{4\left( 1+z_{12}\left( t \right) \right)}-\frac{13}{5}z_{11}\left( t \right)+\zeta_{11}(t) \\ &\frac{dz_{12}\left( t \right)}{dt}=-\frac{2}{5}+\frac{2z_{15}\left( t \right)}{5\left( 1+z_{15}\left( t \right) \right)}+\frac{2}{5\left( 1+z_{16}\left( t \right) \right)}-\frac{14}{5}z_{12}\left( t \right)+\zeta_{12}(t) \\ &\frac{dz_{13}\left( t \right)}{dt}=-\frac{24}{5}+\frac{1}{1+z_{15}\left( t \right)}+\frac{19}{5\left( 1+z_{16}\left( t \right) \right)}-5z_{13}\left( t \right)+\zeta_{13}(t) \\ &\frac{dz_{14}\left( t \right)}{dt}=-\frac{8}{5}+\frac{4}{5\left( 1+z_{10}\left( t \right) \right)}+\frac{4}{5\left( 1+z_{12}\left( t \right) \right)}-\frac{16}{5}z_{14}\left( t \right)+\zeta_{14}(t) \\ &\frac{dz_{15}\left( t \right)}{dt}=\frac{z_{16}\left( t \right)}{10\left( 1+z_{16}\left( t \right) \right)}-\frac{7}{2}z_{15}\left( t \right)+\zeta_{15}(t) \\ &\frac{dz_{16}\left( t \right)}{dt}=\frac{z_{15}\left( t \right)}{10\left( 1+z_{15}\left( t \right) \right)}-\frac{7}{2}z_{16}\left( t \right)+\zeta_{16}(t) \end{aligned} \right.\text{,}\#\text{(}\text{S22}\text{)} \end{aligned}$$

where *q* is a scalar control parameter and $\zeta_{i}(t)$ $(i = 1, 2, \ldots, 16$) are Gaussian noises with zero means. $z_{i}(t)$($i = 1, 2,\ldots,16$) represent the data of gene $i$. In Eq. (S22), the change rates of degradation rates of mRNAs are ($-4\frac{1+q}{15}$, $-2\frac{4+q}{15}$, $-1$, $-\frac{6}{5}$, $-\frac{7}{5}$, $-\frac{8}{5}$, $-\frac{9}{5}$, $-2$, $-\frac{11}{5}$, $-\frac{12}{5}$, $-\frac{13}{5}$, $-\frac{14}{5}$, $-3$, $-\frac{16}{5}$, $-\frac{7}{2}$, $-\frac{7}{2})$. The stable equilibrium point $\bar{Z}=(\bar{z}_{1}, \bar{z}_{2}, \ldots, \bar{z}_{16}) =(0, 0, \ldots, 0)$. The differential equations Eq. (S22) can be transformed into the difference equations $Z\left( k+1 \right)=f(Z\left( k \right),q)$ with a small time interval $\Delta t$ using the Euler scheme [16], *i.e.*,

$$\begin{aligned} \left\{ \begin{aligned} &z_{1}(k+1)=z_{1}\left( k \right)+\left[ \frac{\left( 8-4q \right)z_{2}\left( k \right)}{15\left( 1+z_{2}\left( k \right) \right)}-\frac{4\left( 1+q \right)}{15}z_{1}\left( k \right)+\zeta_{1}\left( k \right) \right]\Delta t \\ &z_{2}(k+1)=z_{2}\left( k \right)+\left[ \frac{(4-2q)z_{1}(k)}{15(1+z_{1}(k))}-\frac{2\left( 1+q \right)}{15}z_{2}\left( k \right)+\zeta_{2}(k) \right]\Delta t \\ &z_{3}(k+1)=z_{3}\left( k \right)+\left[ \frac{4q-10}{15}+\frac{5-2q}{15\left( 1+z_{1}\left( k \right) \right)}+\frac{5-2q}{15\left( 1+z_{2}\left( k \right) \right)}-z_{3}\left( k \right)+\zeta_{3}\left( k \right) \right]\Delta t \\ &z_{4}(k+1)=z_{4}\left( k \right)+\left[ \frac{(6-2q)z_{1}(k)}{15(1+z_{1}(k))}+\frac{(6-2q)z_{2}(k)}{15(1+z_{2}(k))}-\frac{6}{5}z_{4}\left( k \right)+\zeta_{4}(k) \right]\Delta t \\ &z_{5}(k+1)=z_{5}\left( k \right)+\left[ \frac{4q-14}{15}+\frac{7-2q}{15(1+z_{1}(k))}+\frac{7-2q}{15(1+z_{2}(k))}-\frac{7}{5}z_{5}\left( k \right)+\zeta_{5}(k) \right]\Delta t \\ &z_{6}(k+1)=z_{6}\left( k \right)+\left[ \frac{4q-16}{15}+\frac{2(4-2q)}{15(1+z_{1}(k))}+\frac{2(4-2q)}{15(1+z_{2}(k))}-\frac{8}{5}z_{6}\left( k \right)+\zeta_{6}(k) \right]\Delta t \\ &z_{7}(k+1)=z_{7}\left( k \right)+\left[ \frac{(9-2q)z_{1}(k)}{15(1+z_{1}(k))}+\frac{(9-2q)z_{2}(k)}{15(1+z_{2}(k))}-\frac{9}{5}z_{7}\left( k \right)+\zeta_{7}(k) \right]\Delta t \\ &z_{8}(k+1)=z_{8}\left( k \right)+\left[ -\frac{13}{15}+\frac{2}{15\left( 1+z_{1}\left( k \right) \right)}+\frac{2}{15\left( 1+z_{2}\left( k \right) \right)}+\frac{2}{5\left( 1+z_{6}\left( k \right) \right)}+ \right. \\ \left. \frac{2z_{10}\left( k \right)}{5\left( 1+z_{10}\left( k \right) \right)}+\frac{3z_{12}(k)}{5(1+z_{12}(k))}+\frac{z_{15}\left( k \right)}{5\left( 1+z_{15}\left( k \right) \right)}+\frac{1}{5\left( 1+z_{16}\left( k \right) \right)}-2z_{8}\left( k \right)+\zeta_{8}(k) \right]\Delta t \\ &z_{9}(k+1)=z_{9}\left( k \right)+ \\ \left[ -1+\frac{1}{5\left( 1+z_{1}\left( k \right) \right)}+\frac{1}{5\left( 1+z_{2}\left( k \right) \right)}+\frac{3}{5\left( 1+z_{6}\left( k \right) \right)}-\frac{11}{5}z_{9}\left( k \right)+\zeta_{9}(k) \right]\Delta t \\ &z_{10}(k+1)=z_{10}\left( k \right)+\left[ \frac{3z_{12}\left( k \right)}{5\left( 1+z_{12}\left( k \right) \right)}-\frac{12}{5}z_{10}\left( k \right)+\zeta_{10}(k) \right]\Delta t \\ &z_{11}(k+1)=z_{11}\left( k \right)+\left[ \frac{z_{12}\left( k \right)}{4\left( 1+z_{12}\left( k \right) \right)}-\frac{13}{5}z_{11}\left( k \right)+\zeta_{11}(k) \right]\Delta t \\ &z_{12}(k+1)=z_{12}\left( k \right)+\left[ -\frac{2}{5}+\frac{2z_{15}\left( k \right)}{5\left( 1+z_{15}\left( k \right) \right)}+\frac{2}{5\left( 1+z_{16}\left( k \right) \right)}-\frac{14}{5}z_{12}\left( k \right)+\zeta_{12}(k) \right]\Delta t \\ &z_{13}(k+1)=z_{13}\left( k \right)+\left[ -\frac{24}{5}+\frac{1}{1+z_{15}\left( k \right)}+\frac{19}{5\left( 1+z_{16}\left( k \right) \right)}-5z_{13}\left( k \right)+\zeta_{13}(k) \right]\Delta t \\ &z_{14}(k+1)=z_{14}\left( k \right)+\left[ -\frac{8}{5}+\frac{4}{5\left( 1+z_{10}\left( k \right) \right)}+\frac{4}{5\left( 1+z_{12}\left( k \right) \right)}-\frac{16}{5}z_{14}\left( k \right)+\zeta_{14}(k) \right]\Delta t \\ &z_{15}(k+1)=z_{15}\left( k \right)+\left[ \frac{z_{16}\left( k \right)}{10\left( 1+z_{16}\left( k \right) \right)}-\frac{7}{2}z_{15}\left( k \right)+\zeta_{15}(k) \right]\Delta t \\ &z_{16}(k+1)=z_{16}\left( k \right)+\left[ \frac{z_{15}\left( k \right)}{10\left( 1+z_{15}\left( k \right) \right)}-\frac{7}{2}z_{16}\left( k \right)+\zeta_{16}(k) \right]\Delta t \end{aligned} \right.\text{,}\#\text{(}\text{S23}\text{)} \end{aligned}$$

Note that $Z(k)$ is the vector of $Z(t)$ at the time instant $t=k\Delta t$.The Jacobian matrix of Eq. (S22) is denoted as $J=\frac{\partial f(Z;q)}{\partial Z}│_{Z=\bar{Z}}$, where

$$\begin{aligned} J=e^{\Delta t\cdot A}\text{,}\#\text{(}\text{S24}\text{)} \end{aligned}$$

with

$A=\left[ \begin{matrix} \frac{-4-4q}{15} & \frac{8-4q}{15} & 0 & 0 & 0 & 0 & 0 & 0 & 0 & 0 & 0 & 0 & 0 & 0 & 0 & 0 \\ \frac{4-2q}{15} & \frac{-8-2q}{15} & 0 & 0 & 0 & 0 & 0 & 0 & 0 & 0 & 0 & 0 & 0 & 0 & 0 & 0 \\ \frac{-5+2q}{15} & \frac{-5+2q}{15} & -1 & 0 & 0 & 0 & 0 & 0 & 0 & 0 & 0 & 0 & 0 & 0 & 0 & 0 \\ \frac{6-2q}{15} & \frac{6-2q}{15} & 0 & -\frac{6}{5} & 0 & 0 & 0 & 0 & 0 & 0 & 0 & 0 & 0 & 0 & 0 & 0 \\ \frac{-7+2q}{15} & \frac{-7+2q}{15} & 0 & 0 & -\frac{7}{5} & 0 & 0 & 0 & 0 & 0 & 0 & 0 & 0 & 0 & 0 & 0 \\ \frac{-8+2q}{15} & \frac{-8+2q}{15} & 0 & 0 & 0 & -\frac{8}{5} & 0 & 0 & 0 & 0 & 0 & 0 & 0 & 0 & 0 & 0 \\ \frac{9-2q}{15} & \frac{9-2q}{15} & 0 & 0 & 0 & 0 & -\frac{9}{5} & 0 & 0 & 0 & 0 & 0 & 0 & 0 & 0 & 0 \\ -\frac{2}{15} & -\frac{2}{15} & 0 & 0 & 0 & -\frac{2}{5} & 0 & -2 & 0 & \frac{2}{5} & 0 & \frac{3}{5} & 0 & \frac{1}{5} & 0 & -\frac{1}{5} \\ -\frac{1}{5} & -\frac{1}{5} & 0 & 0 & 0 & -\frac{3}{5} & 0 & 0 & -\frac{11}{5} & 0 & 0 & 0 & 0 & 0 & 0 & 0 \\ 0 & 0 & 0 & 0 & 0 & 0 & 0 & 0 & 0 & -\frac{12}{5} & 0 & \frac{3}{5} & 0 & 0 & 0 & 0 \\ 0 & 0 & 0 & 0 & 0 & 0 & 0 & 0 & 0 & 0 & -\frac{13}{5} & \frac{1}{4} & 0 & 0 & 0 & 0 \\ 0 & 0 & 0 & 0 & 0 & 0 & 0 & 0 & 0 & 0 & 0 & -\frac{14}{5} & 0 & 0 & \frac{2}{5} & -\frac{2}{5} \\ 0 & 0 & 0 & 0 & 0 & 0 & 0 & 0 & 0 & 0 & 0 & 0 & -5 & 0 & -1 & \frac{19}{5} \\ 0 & 0 & 0 & 0 & 0 & 0 & 0 & 0 & 0 & \frac{4}{5} & 0 & -\frac{4}{5} & 0 & -\frac{16}{5} & 0 & 0 \\ 0 & 0 & 0 & 0 & 0 & 0 & 0 & 0 & 0 & 0 & 0 & 0 & 0 & 0 & -\frac{7}{2} & \frac{1}{10} \\ 0 & 0 & 0 & 0 & 0 & 0 & 0 & 0 & 0 & 0 & 0 & 0 & 0 & 0 & \frac{1}{10} & -\frac{7}{2} \end{matrix} \right]$.

From Eq. (S24), we obtain sixteen distinct eigenvalues $({0.67}^{q}, 0.45, 0.37, 0.30, 0.25, 0.20, 0.17, 0.14, 0.11, 0.09, 0.07, 0.06, 0.05, 0.04, 0.033, 0.027)$ by taking $\Delta t=1$. It is obvious that ${0.67}^{q}\to1$ as $q\to0$, indicating that there is a critical value $q_{c}=0$, where the system undergoes a critical transition. We aimed to detect early warning signals that indicate the critical transition as a control parameter $q$ approaches a critical value 0. It should be noted that the kinetic model has nothing to do with the application on real biological data. Model Eq. (S22) is only for a numerical simulation that is totally irrelevant with the real-data applications.

## Supplementary Note S7. Details for the expression calculation of DEGs

The average expression of differentially expressed genes (DEGs) represents the aggregate of expression of DEGs across all samples within a time point $T=t$. Specifically, for the gene expression matrix of case samples at time point $T=t$, we conducted differential analysis by comparing them with the reference (relatively normal) samples, generating a set of DEGs ($P<0.05$) at time point $T=t$, denoted as $D^{t}$. Subsequently, we retained the top 5% significantly differentially expressed genes from $D^{t}$, denoted as $D_{top}^{t}$. The size of $D_{top}^{t}$ is denoted as $\alpha^{t}$. The case sample set at time point $T=t$ is denoted as $C^{t}$ and its size is denoted as $\beta^{t}$. Next, we computed the average value of the significantly differentially expressed genes across all samples at time point $T=t$ as follows:

$$\begin{aligned} v^{t}=\frac{1}{\alpha^{t}\beta^{t}}\sum_{g\in D_{top}^{t},k\in C^{t}} \rho_{gk}^{t}\text{,}\#\text{(}\text{S25}\text{)} \end{aligned}$$

where $\rho_{gk}^{t}$ represents the expression value of a gene $g\in D_{top}^{t}$ in a case sample $k\in C^{t}$ at time point $T=t$. Finally, we repeated the aforementioned steps across all time points to generate the average expression data lines for DEGs.

## Supplementary Note S8. Verification for the identified critical state

Inspired by other study [25], we employed a procedure illustrated in Fig. S11 to verify the identified critical state. For example, if BCTI determines that the critical stage is IIA, then the first survival test compares samples from the before-transition period (IA-IIA) with those from the after-transition period (IIIB-IV), the second survival test compares any two stages from the before-transition except for the critical stage, the third survival test compares any two stages from the after-transition except for the critical stage, and the fourth survival test compares the before-transition period (IA-IIA) with the following stage of the critical stage (IIB). In addition, when survival test 1 and test 4 have a significant difference in survival time, it indicates that the survival time of the samples exhibit a dramatic shift following the critical stage. Furthermore, when test 2 and test 3 have a negligible difference in survival time, it indicates that there are no significant differences in survival status among patients both before and after the identified critical stage. A schematic illustration for the above strategy is displayed in Fig. S11.

Taking THCA as an example (Fig. S12), BCTI identified stage II as the critical state for THCA. Using the above validation strategy, we found a significant difference in the survival time between samples from the before-transition state (I–II) and those from the after-transition state (III–IV) ($P<0.0001$; Fig. S12A). This indicates that the survival time of patients in the stages following the critical shift is shorter than that of patients in the stages before the transition. Furthermore, survival analysis of after-transition samples was conducted to determine if there were any other significant transitions affecting survival time. As a result, the survival curves for samples in stages III and IV showed no significant difference ($P=0.35$; Fig. S12B), indicating that there were no other significant transitions affecting survival time. At last, we performed survival analysis for before-transition samples (at stage I-II) and the samples at stage III. It is found that samples in stages I–II exhibit a markedly longer survival duration compared to those in stage III ($P=0.0016$; Fig. S12C), indicating a dramatic shift in survival status following the critical stage. To sum up, the above results validated the effectiveness of BCTI in detecting the critical point during disease progression.

# Supplementary references

1. Buljan M, Blattmann P, Aebersold R et al. Systematic characterization of pan‐cancer mutation clusters. *Molecular systems biology* 2018;14:e7974.

2. Chen Y, Wang K, Shang M et al. Exploration of DNA methylation-driven genes in papillary thyroid carcinoma based on the cancer genome atlas. *Journal of Computational Biology* 2021;28:99-114.

3. Wang S, Wang H, Liu J et al. Expression patterns and functional analysis of porcine lnc-34015. *Animal Biotechnology* 2023;34:2251-2261.

4. Hong Y, Heo J, Kang S et al. Exosome-mediated delivery of gga-miR-20a-5p regulates immune response of chicken macrophages by targeting IFNGR2, MAPK1, MAP3K5, and MAP3K14. *Animal bioscience* 2023;36:851.

5. Li H, Duan N, Zhang Q et al. IL1A & IL1B genetic polymorphisms are risk factors for thyroid cancer in a Chinese Han population. *International Immunopharmacology* 2019;76:105869.

6. Untch BR, Dos Anjos V, Garcia-Rendueles ME et al. Tipifarnib inhibits HRAS-driven dedifferentiated thyroid cancers. *Cancer research* 2018;78:4642-4657.

7. Chen P, Li Y, Liu X et al. Detecting the tipping points in a three-state model of complex diseases by temporal differential networks. *Journal of translational medicine* 2017;15:1-15.

8. Scheffer M, Carpenter S, Foley JA et al. Catastrophic shifts in ecosystems. *Nature* 2001;413:591-596.

9. Chen L, Liu R, Liu Z-P et al. Detecting early-warning signals for sudden deterioration of complex diseases by dynamical network biomarkers. *Scientific reports* 2012;2:342.

10. Basso K, Margolin AA, Stolovitzky G et al. Reverse engineering of regulatory networks in human B cells. *Nature genetics* 2005;37:382-390.

11. Rochon J, Kieser M. A closer look at the effect of preliminary goodness‐of‐fit testing for normality for the one‐sample t‐test. *British Journal of Mathematical and Statistical Psychology* 2011;64:410-426.

12. Huynh-Thu VA, Irrthum A, Wehenkel L et al. Inferring regulatory networks from expression data using tree-based methods. *PloS one* 2010;5:e12776.

13. Wu J, Zhao X, Lin Z et al. Large scale gene regulatory network inference with a multi-level strategy. *Molecular Biosystems* 2016;12:588-597.

14. Zhang Y, Chang X, Liu X. Inference of gene regulatory networks using pseudo-time series data. *Bioinformatics* 2021;37:2423-2431.

15. Iglesias-Martinez LF, De Kegel B, Kolch W. KBoost: a new method to infer gene regulatory networks from gene expression data. *Scientific reports* 2021;11:15461.

16. Zhang X, Liu K, Liu Z-P et al. NARROMI: a noise and redundancy reduction technique improves accuracy of gene regulatory network inference. *Bioinformatics* 2013;29:106-113.

17. Ruyssinck J, Huynh-Thu VA, Geurts P et al. NIMEFI: gene regulatory network inference using multiple ensemble feature importance algorithms. *PloS one* 2014;9:e92709.

18. Guo S, Jiang Q, Chen L et al. Gene regulatory network inference using PLS-based methods. *BMC bioinformatics* 2016;17:1-10.

19. Liu R, Chen P, Chen L. Single-sample landscape entropy reveals the imminent phase transition during disease progression. *Bioinformatics* 2020;36:1522-1532.

20. Chen L, Wang R, Li C et al. Modeling biomolecular networks in cells: structures and dynamics. Springer Science & Business Media, 2010.

21. Zomaya AY, Pan Y. Biomolecular networks: methods and applications in systems biology. John Wiley & Sons, 2009.

22. Becskei A, Serrano L. Engineering stability in gene networks by autoregulation. *Nature* 2000;405:590-593.

23. Chen L, Aihara K. Stability of genetic regulatory networks with time delay. *IEEE Transactions on circuits and systems I: Fundamental Theory and Applications* 2002;49:602-608.

24. Li C, Chen L, Aihara K. Stability of genetic networks with SUM regulatory logic: Lur'e system and LMI approach. *IEEE Transactions on Circuits and Systems I: Regular Papers* 2006;53:2451-2458.

25. Zhong J, Liu R, Chen P. Identifying critical state of complex diseases by single-sample Kullback–Leibler divergence. *BMC genomics* 2020;21:1-15.
